# Analysis of *Vibrio harveyi* adaptation in sea water microcosms at elevated temperature provides insights into the putative mechanisms of its persistence and spread in the time of global warming

**DOI:** 10.1038/s41598-018-36483-0

**Published:** 2019-01-22

**Authors:** Itxaso Montánchez, Elixabet Ogayar, Ander Hernández Plágaro, Anna Esteve-Codina, Jèssica Gómez-Garrido, Maite Orruño, Inés Arana, Vladimir R. Kaberdin

**Affiliations:** 1 Department of Immunology, Microbiology and Parasitology, University of the Basque Country UPV/EHU, 48940 Leioa, Spain; 2 CNAG-CRG, Centre for Genomic Regulation (CRG), Barcelona Institute of Science and Technology (BIST), Barcelona, Catalonia 08028 Spain; 3 Universitat Pompeu Fabra (UPF), Barcelona, Catalonia 08003 Spain; 4 Research Centre for Experimental Marine Biology and Biotechnology (PIE-UPV/EHU), 48620 Plentzia, Spain; 5 IKERBASQUE, Basque Foundation for Science, Maria Diaz de Haro 3, 48013 Bilbao, Spain

## Abstract

Discovering the means to control the increasing dissemination of pathogenic vibrios driven by recent climate change is challenged by the limited knowledge of the mechanisms in charge of *Vibrio* spp. persistence and spread in the time of global warming. To learn about physiological and gene expression patterns associated with the long-term persistence of *V*. *harveyi* at elevated temperatures, we studied adaptation of this marine bacterium in seawater microcosms at 30 °C which closely mimicked the upper limit of sea surface temperatures around the globe. We found that nearly 90% of cells lost their culturability and became partly damaged after two weeks, thus suggesting a negative impact of the combined action of elevated temperature and shortage of carbon on *V*. *harveyi* survival. Moreover, further gene expression analysis revealed that major adaptive mechanisms were poorly coordinated and apparently could not sustain cell fitness. On the other hand, elevated temperature and starvation promoted expression of many virulence genes, thus potentially reinforcing the pathogenicity of this organism. These findings suggest that the increase in disease outbreaks caused by *V*. *harveyi* under rising sea surface temperatures may not reflect higher cell fitness, but rather an increase in virulence enabling *V*. *harveyi* to escape from adverse environments to nutrient rich, host-pathogen associations.

## Introduction

*Vibrio harveyi* is a heterotrophic Gram-negative luminous bacterium inhabiting marine environments and showing a preference for temperate and tropical waters. In its natural habitats, *Vibrio* spp. can be found attached to either biotic or abiotic surfaces^1^, in a free living state as well as in symbiotic^2^ or host-pathogen^3^ interactions with other organisms, occasionally causing diseases or even death of the infected organisms^4^.

The most recent studies have revealed a wider spread of pathogenic vibrios and this phenomenon was documented along with a gradual increase in sea surface temperature taking place in the time of global warming (GW)^5^. Consistent with the above trends, there have been a concomitant increase in the cholera or/and gastroenteritis outbreaks^6–8^ apparently caused by the consumption of water contaminated with pathogenic vibrios^9–12^. As the average temperature of water increases towards the equator, more cases of *Vibrio*-related diseases were reported in countries such as India or those in the Caribbean^13,14^. Moreover, a number of recent reports clearly point to the emergence of *Vibrio*-associated diseases in Europe including some countries where the presence of virulent variants of *V*. *vulnificus* and other pathogenic strains could barely been detected previously^15^. This alarming statistics includes discovery of pathogenic *Vibrio* species in several unexpected locations such as finding of *V*. *cholerae* in estuaries of Portugal^16^, *V*. *parahaemolyticus* on the French Atlantic coast^17^ and the highly pathogenic strain *V*. *parahaemolyticus serovar* O3:K6 in Spain^18–21^.

In accordance with the overall increase in the occurrence of *Vibrio*-associated diseases^22^, several highly virulent *V*. *harveyi* strains able to provoke mass mortality in both marine invertebrates^23,24^ and various fishes^25,26^ have been reported as well. *V*. *harveyi* is notoriously known for being an important pathogen of cultured penaeid shrimp^27^ and its wider occurrence in aquaculture farms causes serious problems for the seafood industry, especially in tropical countries where temperatures are generally higher. As the forecast is not promising, it is conceivable that the ocean is going to experience even more devastating consequences of GW in the coming years, which could likely lead to further spread of *Vibrio*-associated infections.

The ubiquitous presence and further spread of *Vibrio* spp. indicate that these marine bacteria are able to easily adapt to changing environmental conditions including those caused by GW. Several studies have previously examined their adaptation by analyzing phenotypical and gene expression changes taking place in *Vibrio* species facing different stress conditions^28,29^. Analysis of *V*. *harveyi* adaptation revealed that deprivation of nutrients (starvation) can trigger profound morphological changes leading to reduction of cell size and conversion of rod-shaped bacteria into their coccoid-like variants^30–32^. In addition, when environmental conditions become unfavorable (e.g. limitation of nutrients, low temperature, low salinity or ultra violet (UV) radiation), *V*. *harveyi* can occasionally acquire the viable but nonculturable (VBNC) phenotype^33,34^ and preserve it until the adverse conditions are eliminated, subsequently being able to recover from this state of dormancy and resume growth.

An important characteristics that enables vibrios to successfully strive in marine ecosystems is their metabolic versatility, in particular, their capacity to use a wide variety of organic substances (e.g. D-glucose, N-acetyl-D-glucosamine, L-asparagine aconitate *etc*.) as a primary carbon source. Moreover, the use of different carbon sources is, in turn, facilitated by the ability of *Vibrio* spp. to secrete various hydrolytic enzymes (amylases, gelatinases, lipases and chitinases) converting the naturally occuring biopolymers (e.g. proteins, polysaccharides, chitin *etc*.) into smaller, easily metabolizable compounds^35^.

Although changing environments are known to have a great impact on marine bacteria, little is known about how the physiology, metabolic preferences and adaptation mechanisms of *Vibrio* spp. are affected by the main environmental factors (elevated temperature, ocean acidification, suboptimal salinity concentration, *etc*.) caused by the ongoing GW. In order to learn more about *Vibrio* spp. responses to GW and their possible contribution to survival and pathogenicity of *Vibrio* spp., we studied phenotypical and gene expression changes that occur during the time-dependent adaptation of *V*. *harveyi* in seawater microcosm at 30 °C which is close to the upper limit of sea surface temperature (SST) recently observed in some regions of the globe^36,37^.

### Experimental procedures

#### Survival assays, bacterial cell count and size measurements

To assess the combined effect of carbon limitation and elevated temperature on physiology and appearance of *Vibrio harveyi* ATCC® 14126™, cells were aerobically grown overnight at 26 °C (optimal temperature for *V*. *harveyi* growth) in Marine broth (MB) (Panreac) and three 20 mL aliquots of the stationary phase cultures were independently diluted (1:20) with sterile natural seawater to obtain 400 mL suspensions with a final density of 10^8^ cells mL^−1^. The resulting inoculates of *V*. *harveyi* were incubated in dark with shaking (90 rpm) at 30 °C within 21 days. The experiments were carried out in 1 L glass flasks beforehand cleaned with H_2_SO_4_ (97%, v/v), rinsed with deionized water and heated at 250 °C for 24 h to avoid any presence of residual organic matter.

The seawater was sampled in the Port of Armintza in the Bay of Biscay, 43°26′24″N and 2°54′24″W and then was filter sterilized and autoclaved. The concentration of dissolved organic carbon (DOC) previously measured in the same area is in the range of 0.2–0.4 mM^38^, which is nearly 200 times lower than the concentration of organic carbon in MB (https://www.nature.com/articles/nrmicro1752/tables/1).

Periodically, samples were collected for further analysis. The total number of cells was determined by filtering aliquots of *V*. *harveyi* suspensions (withdrawn after 3, 6, 9, 13 and 21 days of incubation) through 0.22 μm pore-size polycarbonate membrane filters (Millipore), followed by staining of the attached cells with acridine orange and direct counting individual cells using epifluorescence microscopy^39^. The microscope (Eclipse E400, Nikon) was equipped with a video camera Hamamatsu 2400 (Hamamatsu Photonics, Japan) enabling to obtain high resolution images for their subsequent analysis by Scion Image 1.62^a^ software to determine the size of individual cells as described by^40^. Approximately 150–200 cells were measured in each sample, and, according to their length, the cells fell into three groups:≤1.2 µm, >1.2–2 µm and >2 µm^32^.

Cultivability expressed as colony-forming units (CFU) was evaluated by spreading aliquots (withdrawn after 2, 3, 5, 7, 9, 12, 15, 17, 19 and 21 days of incubation) obtained by consecutive dilutions of *V*. *harveyi* suspensions on Marine Agar (MA, Oxoid) followed by incubation for 24 h at 26 °C.

#### Scanning electron microscopy

*V*. *harveyi* cells present in the control (overnight culture) and test samples (i.e after 3, 6 and 21 days of incubation in seawater at 30 °C) were fixed by adding 50 µl of 3% formalin to each sample (1 mL) and were further stored at 4 °C. The suspensions of the fixed cells were filtered through 0.22 μm pore-size membrane filters (GTTP filters, Millipore) and the cells attached to the filters were further examined by scanning electron microscopy (SEM) at the Advance Research Core Facility Unit (SGIker) of the University of the Basque Country. Briefly, the filters with the attached *V*. *harveyi* cells were dehydrated by sequentially immersing them (for 5 min each time) in water/ethanol solutions containing increasing concentrations of ethanol (30, 50, 70, 90, and 100%, respectively) followed by overlaying with hexamethyldisilazane, incubation for 5 min and drying on air. Finally, the samples were coated with a layer of 15 nm gold by using an Emitech K550X Sputter Coater, and imaging was further carried out by examining samples in a Hitachi S4800 scanning electron microscope.

#### RNA isolation, sequencing and processing of RNAseq data

*V*. *harveyi* ATCC® 14126™ cultures were grown in triplicate overnight (12–16 h) at 26 °C in MB, and 20 mL of each culture were individually diluted (1:20) with sterile seawater (originated from the same batch of sterile seawater used in the survival assays) and further incubated in 1 L glass flasks with shaking (90 rpm) at 30 °C. Aliquots of *V*. *harveyi* suspensions were withdrawn after 12 h, 3 and 6 days of incubation, mixed (8:1) with stop solution (5% phenol in ethanol) and then incubated on ice for 15–20 min. The cells were collected by centrifugation (15 min, 4 °C, 4400 g) and the resulting cell pellets were further used to isolate total RNA by employing TRIzol reagent and PureRNA mini Kit (Invitrogen) following the vendor’s instructions.

After verifying the quality of the purified RNA by using Bioanalyzer (Agilent), it was further processed with the assistance of the General Genomic Service (SGIker) of the University of the Basque Country. The libraries were prepared from total RNA by using ScriptSeq Complete kit (Illumina) optimized for bacterial RNA. The complete kit included the Ribo-Zero rRNA Removal Kit and ScriptSeq v2 Kit with ScriptSeq Index PCR Primers Set. The Ribo-Zero rRNA Removal kit was used to deplete 275–400 ng of total RNA with ribosomal RNA following the low input protocol. The samples depleted with rRNA were then concentrated with RNA Clean & Concentrator-5 kit (Zymo Research) for recovery and preservation of large RNA fragments (>200 nt) and afterwards were used to prepare stranded RNA-Seq libraries according to the manufacturer’s instructions. Further RNA sequencing and bioinformatics analysis were carried out at the National Center for Genomic Analysis (CNAG) in Barcelona integrated with the Center for Genomic Regulation (CRG). The libraries were sequenced on HiSeq. 4000 (Illumina) in a paired-end mode with a read length of 2 × 76 bp using HiSeq. 4000 SBS kit in a fraction of a HiSeq. 4000 PE Cluster kit sequencing flow cell lane following the standard protocol.

Image analysis, base calling and quality scoring of the run were processed using the manufacturer’s software Real Time Analysis (RTA 2.7.6) and followed by generation of FASTQ sequence files by CASAVA. The processed RNA-seq reads were mapped against *V*. *harveyi* ATCC® 43516™ reference genome (available at ftp://ftp.ncbi.nlm.nih.gov/genomes/genbank/; accession number GCA_001558435.1) with STAR^41^ and were further quantified with RSEM^42^. Information about some sRNAs initially absent in the annotation of *V*. *harveyi* ATCC® 43516™ genome was added to the annotation file by performing a highly restrictive BLAST search by using the sequences of *V*. *campbellii* ATCC® BAA-1116™ sRNAs retrieved from RFAM and BSRD databases as queries. Functional annotation of other genes was performed by blasting each assembled gene sequence to find homology to other V. *harveyi* genes already annotated in the NCBI database and the functional annotations of the best hits was used for the final annotations of transcripts revealed by RNA-seq. Differential expression analysis was performed with DESeq. 2 R^43^ and genes with false discovery rate (FDR)<5% and fold change >2 were considered significant and were further used to construct temporal clusters. Time series cluster analysis according to the soft clustering algorithm was performed with Mfuzz bioconductor package^44^ with k = 6 found to be optimal to obtain the minimal average cluster overlap. Gene ontology enrichment of the differentially expressed genes was performed with GOstats^45^. All raw and processed RNA-seq data were deposited into the GEO archive (https://www.ncbi.nlm.nih.gov/geo/) and are publicly available under accession number GSE113564.

## Results

### Effects of prolonged incubation of *V*. *harveyi* cells in seawater at 30 °C on their culturability and morphology

**Figure 1:**
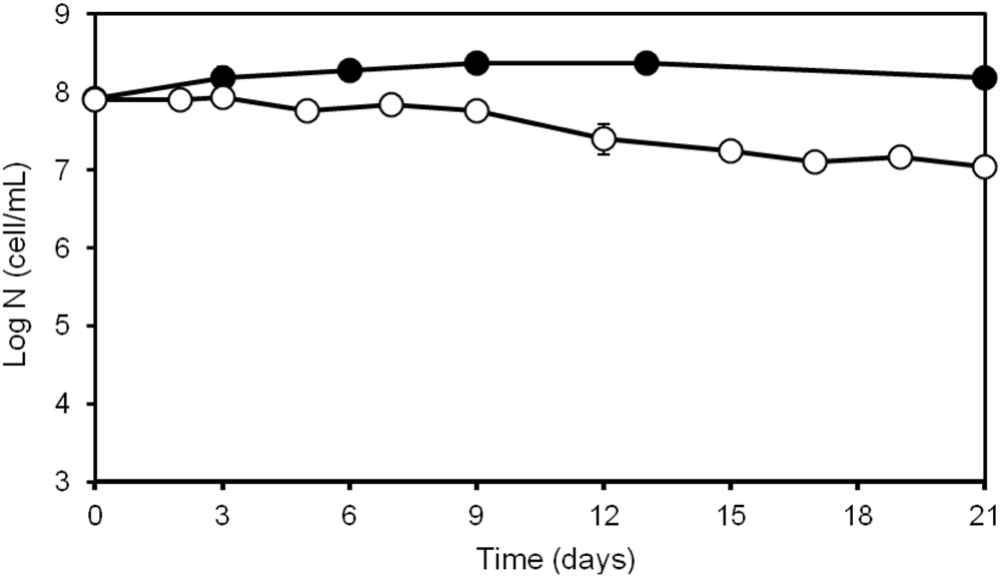
*Vibrio harveyi* counts obtained during its persistence in seawater at 30 °C. The number of total (•) and culturable (○) bacteria were estimated as described in Materials and Methods. The data are mean values from three independent experiments with errors bars representing the standard deviations calculated.

Experiments were done with three independent microcosms (biological replicates) prepared by diluting aliquots of the stationary phase cultures (control) with sterile seawater and analyzing the appearance and culturability of *V*. *harveyi* populations after 12 hours, 3, 6, 14 and 21 days of incubation. While determining culturable populations in aliquots withdrawn at different time points and analyzed by spreading on MA plates, a 10-fold decrease in the number of culturable cells was observed after three weeks of incubation (Fig. 1).

**Figure 2:**
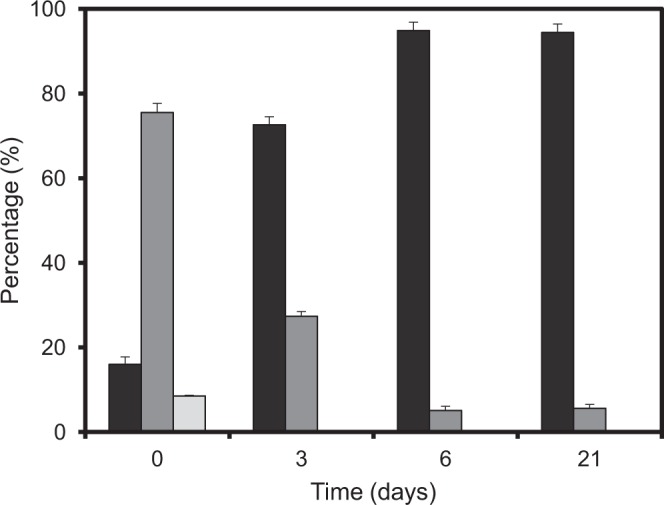
Size distribution of *Vibrio harveyi* cells during their persistence in seawater at 30 °C. The values were obtained by analyzing cell images captured by epifluorescence microscopy and processed as described in Materials and Methods. The bars show the percentage of cell within each size range (■ ≤1.2 µm;  >1.2 − ≤ 2 µm and  >2 µm, respectively).

Moreover, the loss of culturability was accompanied by a marked reduction of cell size that became readily distinguishable already after 3 days of incubation. Furthermore, a gradual increase of the fractions of smaller cells was observed until the last day of the measurements (Fig. 2). In other words, while 75.5% of the cells in the control sample represented by the overnight culture (day 0) had size between 1.2 to 2 µm, this number has decreased to 27.4% after 3 days of incubation and the percentage of longer cells continued decreasing progressively until day 6, when nearly all cells (i.e. 95%) were shorter than 1.2 µm.

**Figure 3:**
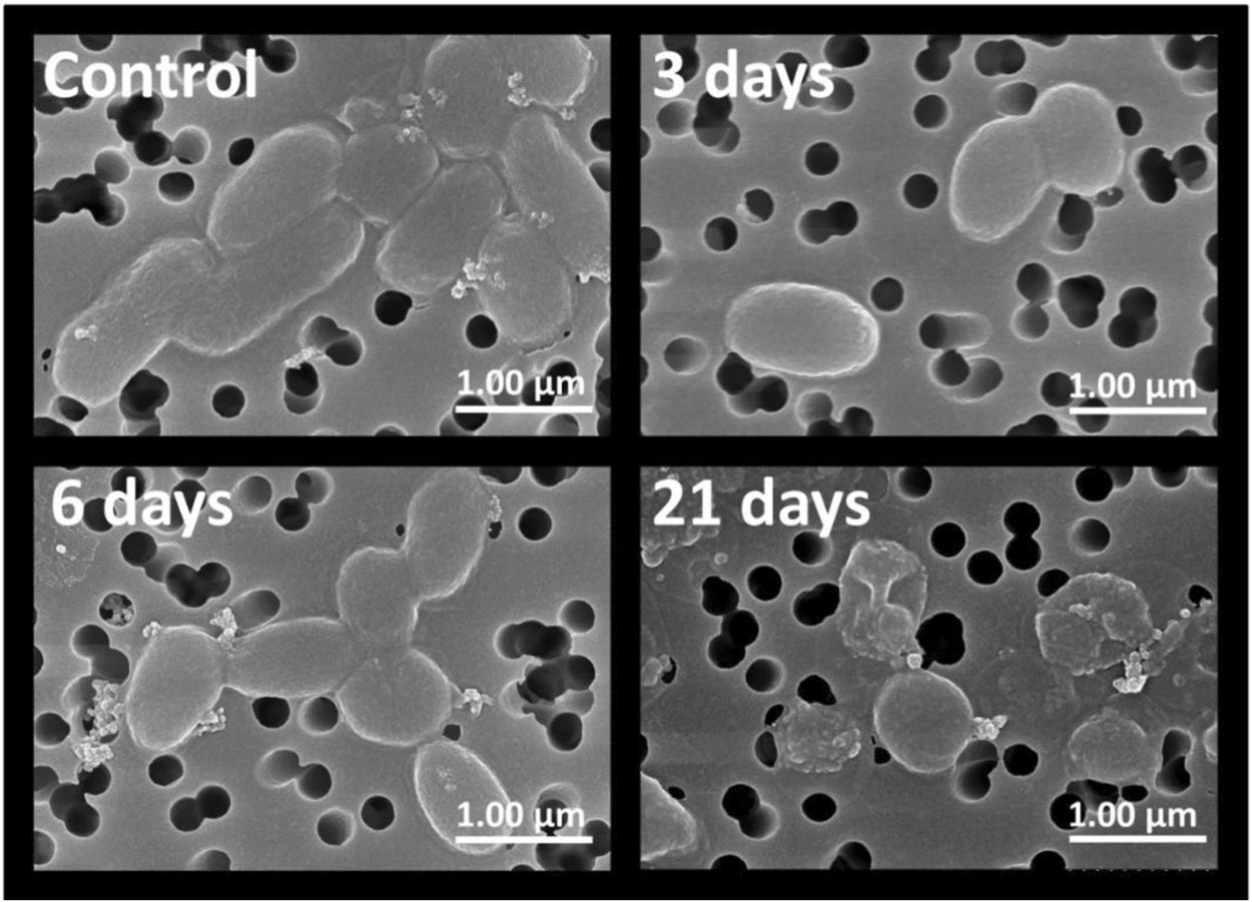
Morphology and integrity of *Vibrio harveyi* populations incubated in seawater at 30 °C. Scanning electron microscopy images were obtained for the cells present in the initial inoculate (control) as well as those incubated at 30 °C for 3, 6 and 21 days, respectively.

The observed size reduction was further corroborated by the results of scanning electron microscopy (SEM). SEM confirmed (Fig. 3) the appearance of cells with a coccoid-like morphology (i.e. with a nearly spherical shape) after 3 to 6 days of incubation. Moreover, the electron microscopy revealed a considerable number of damaged cells, thus indicating that prolong incubation (>6 days) under carbon limitation at 30 °C can lead to the loss of cell integrity.

### Gene expression analysis

Besides monitoring cell culturability and morphological changes, *V*. *harveyi* adaptation was also analyzed at the whole transcriptome level. To carry out this analysis, total RNA was isolated from the initial inoculate (control) and from *V*. *harveyi* cells after their incubation in sterile seawater for 12 h, 3, 6, 14 and 21 days. Total RNA samples were extracted from replicate cultures, i.e. they were originated from three independent cell cultures (biological replicates) grown in parallel. Electrophoretic profiles of RNA samples isolated from cells incubated for 14 and 21 days revealed that they contained a considerable fraction of degraded RNA (apparently accumulating due to cell damage beginning after prolong incubation of *V*. *harveyi* cells in seawater (Fig. 3). RNA purified from the control (t = 0) and microcosms at early time points (12 h, 3 and 6 days) were used for RNA sequencing.

**Figure 4:**
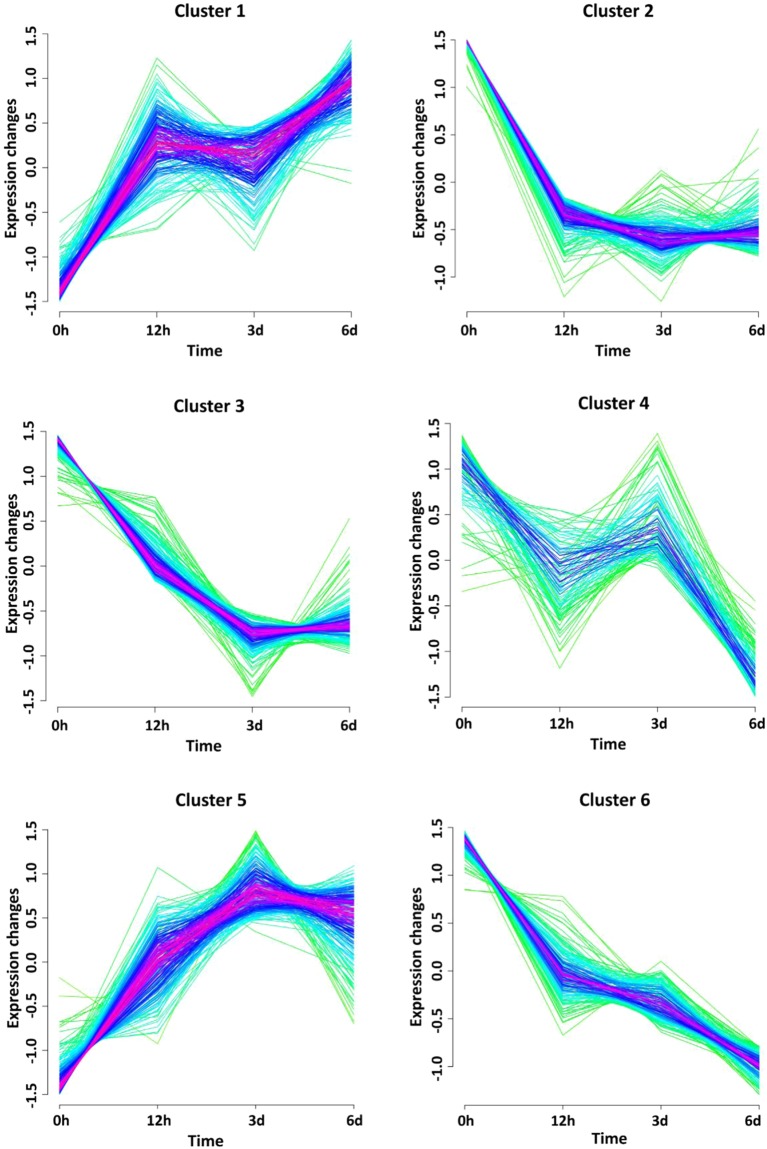
Gene co-expression clusters. Temporal expression patterns were obtained for the time-points corresponding to *Vibrio harveyi* incubation in seawater at 30 ^o^C for 12 hours, 3 days and 6 days. Gene membership values are color-encoded with red and green shades denoting high and low membership values of genes, respectively. Scaled expression values (z-scores) are used for cluster visualization.

Based on the results of RNA sequencing and their processing (see Materials and Methods), a list of the differentially expressed genes with false discovery rate less than 5% and fold change >2 was compiled (see Table S1). Further bioinformatics analysis of this list made it possible to reveal six clusters of genes with similar expression profiles (Fig. 4). Two of them (clusters 1 and 5) were gradually upregulated during the course of the experiment and included a number of genes (see Table 1) primarily related to transport, virulence (clusters 1 and 5) and stress responses (cluster 5). The other four clusters (i.e. clusters 2, 3, 4 and 6) contained numerous genes controlling various cellular functions. The expression of these genes either was initially decreased and then remained low (clusters 2, 3 and 6) or was transitionally upregulated (cluster 4) at later time points. While clusters 2 and 3 were primarily represented by metabolic genes (e.g. genes controlling carboxylic acid and nucleotide metabolism, respectively), the other two (clusters 4 and 6) comprised the genes playing important roles in the processing of genetic information (namely, transcription and translation, respectively).

**Table 1:** Some examples of *V*. *harveyi* genes and their products highly up- or down-regulated during persistence in seawater microcosm at 30 °C.

Systematic name	Gene product (specific biological pathway)	Log_2_ Fold change (time after exposure to seawater)			Gene expression cluster
		12 h	3 d	6 d	
**Amino acid metabolism**					
AL538_RS23670	Methylmalonate-semialdehyde dehydrogenase (CoA acylating)	−2.325	−3.377	−2.733	2
AL538_RS15645	Alanine dehydrogenase	−2.877	−2.910	−3.289	2
AL538_RS01790	Ornithine cyclodeaminase	−2.714	−4.302	−3.209	2
AL538_RS07820	Argininosuccinate synthase	−2.225	−2.890	−2.258	2
AL538_RS07290	Aspartate carbamoyltransferase	−1.401	−1.345	−1.897	6
AL538_RS07295	Aspartate carbamoyltransferase regulatory subunit	−1.251	−1.976	−1.289	2
AL538_RS25000	Glutamate synthase	−0.858	−1.233	−1.597	6
AL538_RS11745	Glutamate synthase subunit beta	−1.595	−2.359	−1.828	2
AL538_RS01370	Alkaline serine protease	−1.378	−3.486	−2.372	4
AL538_RS14635	Serine protein kinase PrkA	−1.385	−1.333	−2.215	6
AL538_RS01665	4-hydroxyphenylpyruvate dioxygenase	−2.624	−2.794	−3.415	2
AL538_RS03245	Ribonucleotide-diphosphate reductase subunit beta	−1.553	−2.794	−2.503	3
AL538_RS14510	Uridine phosphorylase	−1.682	−2.946	−3.119	3
**Carbon metabolism**					
**Citrate, glyoxylate & 2-methylcitrate cycles**					
AL538_RS17025	Citrate synthase/methylcitrate synthase	−2.020	−1.656	−2.315	6
AL538_RS06380	Aconitate hydratase B	−1.588	−2.634	−2.372	3
AL538_RS10235	Phosphoenolpyruvate carboxykinase (ATP)	−1.458	−3.015	−2.504	3
**Glycolysis and gluconeogenesis**					
AL538_RS06930	Phosphoglycerate kinase	−1.341	−2.545	−2.503	3
AL538_RS10235	Phosphoenolpyruvate carboxykinase (ATP)	−1.458	−3.015	−2.504	3
AL538_RS04660	Type I glyceraldehyde-3-phosphate dehydrogenase	−1.325	−2.871	−2.660	3
**Lipid biogenesis**					
AL538_RS16785	Lipid A biosynthesis lauroyl acyltransferase	0.664	0.993	1.203	1
AL538_RS25885	Choline dehydrogenase	1.176	1.370	0.993	5
AL538_RS03425	Acyl-CoA thioesterase	0.878	1.704	1.073	5
AL538_RS06930	Phosphoglycerate kinase	−1.341	−2.545	−2.503	3
AL538_RS26865	Glycerophosphoryl diester phosphodiesterase	−2.629	−3.534	−2.947	2
AL538_RS23675	Acyl-CoA dehydrogenase	−2.053	−2.768	−2.308	2
AL538_RS23680	Enoyl-CoA hydratase	−2.034	−2.947	−2.470	2
AL538_RS27060	Beta-ketoacyl-ACP reductase	−1.347	−2.564	−2.983	3
AL538_RS03900	Malonyl CoA-acyl carrier protein transacylase	−1.815	−2.683	−2.368	3
**Acetyl-CoA-dependent metabolism**					
AL538_RS14965	Peptidoglycan-associated lipoprotein	−1.663	−2.943	−2.801	3
AL538_RS04550	Bifunctional acetaldehyde-CoA/alcohol Dehydrogenase	−1.470	−3.161	−2.871	3
AL538_RS08465	Acetyl-CoA synthetase	−1.601	−2.312	−2.177	3
**Processing of genetic information**					
AL538_RS06035	Elongation factor G	−1.316	−2.547	−2.559	3
AL538_RS12075	Energy-dependent translational throttle protein EttA	−1.550	−2.247	−2.244	3
AL538_RS02125	Translation initiation factor IF-3	−1.436	−2.093	−2.476	6
**Ribosome biogenesis**					
AL538_RS07900	30S ribosomal protein S12	−1.454	−2.108	−2.716	6
AL538_RS10850	50S ribosomal protein L22	−0.954	−2.066	−2.065	6
AL538_RS03245	Ribonucleotide-diphosphate reductase subunit beta	−1.553	−2.794	−2.503	3
**Transporters and ancillary factors**					
**Energy-dependent transport TonB-ExbB-ExbD complex**					
AL538_RS26550	Biopolymer transporter ExbB	1.623	2.108	1.740	5
AL538_RS19445	TonB-system energizer ExbB	0.201	1.186	1.025	5
**ABC transporters**					
AL538_RS26870	ABC transporter substrate-binding protein	−2.683	−4.749	−3.279	2
AL538_RS19380	Amino acid ABC transporter	−1.236	−1.698	−1.907	3
AL538_RS00950	Amino acid ABC transporter substrate-binding protein	−1.188	−2.888	−2.524	3
AL538_RS01680	Peptide ABC transporter substrate-binding protein	−2.146	−4.524	−3.021	3
AL538_RS01780	Polyamine ABC transporter ATP-binding protein	−2.676	−3.963	−3.018	2
AL538_RS01795	Spermidine/putrescine ABC transporter substrate-binding protein	−2.751	−4.780	−3.382	2
**Other transporters**					
AL538_RS01055	EamA family transporter	0.950	1.603	1.870	5
AL538_RS25015	Long-chain fatty acid transporter	0.640	0.909	1.212	1
AL538_RS21850	MFS transporter	1.249	1.445	1.537	1
AL538_RS17670	PTS mannitol transporter subunit IIA	1.036	1.597	1.373	5
**Protein turnover and folding**					
**Protein degradation**					
AL538_RS01370	Alkaline serine protease	−1.378	−3.486	−2.372	4
AL538_RS12810	Aminoacyl-histidine dipeptidase	−1.960	−2.991	−2.215	2
AL538_RS10020	Oligopeptidase A	−1.105	−1.895	−2.198	6
AL538_RS01150	Peptidase A24	0.249	1.061	1.581	1
AL538_RS01675	Peptidase M20	0.859	0.886	1.368	1
AL538_RS24140	Peptidase M50	1.118	0.888	1.354	1
AL538_RS25255	Peptidase S8	0.698	0.813	1.005	1
AL538_RS18135	D-alanyl-D-alanine carboxypeptidase	−1.318	−2.530	−2.336	3
AL538_RS01705	Xaa-Pro aminopeptidase	−2.220	−4.042	−3.019	3
**Protein folding**					
AL538_RS25315	Molecular chaperone	0.594	1.004	1.011	5
AL538_RS05430	Molecular chaperone	−1.777	−1.784	−2.056	2
AL538_RS00890	RNA chaperone ProQ	−1.198	−1.487	−2.016	6
**Energy production**					
AL538_RS03195	Cytochrome c nitrite reductase subunit NrfD	0.565	1.438	0.764	5
AL538_RS00765	Cytochrome-c oxidase, cbb3-type subunit III	−1.764	−2.531	−2.308	2
**ATP synthesis coupled proton transport**					
AL538_RS09605	F0F1 ATP synthase subunit delta	−1.768	−3.170	−2.672	3
AL538_RS09595	F0F1 ATP synthase subunit gamma	−1.825	−3.548	−2.778	3
**Fumarate reductase (complex II)**					
AL538_RS08265	Fumarate reductase flavoprotein subunit	−1.862	−2.041	−1.584	2
AL538_RS08280	Fumarate reductase subunit D	−1.508	−2.045	−1.847	2
**Stress responses**					
**Cell envelope stress: Phage-shock-protein response**					
AL538_RS15985	Phage shock protein PspA	−0.908	0.819	−1.526	4
AL538_RS15995	Envelope stress response membrane protein PspC	−1.042	−0.263	−1.819	4
**Stringent response**					
AL538_RS11565	Stringent starvation protein B	−1.121	−0.789	−1.168	2
**Antioxidative defence**					
AL538_RS17640	Catalase	1.260	2.075	1.668	5
AL538_RS21845	Glutaredoxin, GrxB family	0.713	1.418	0.700	5
**Nitric oxide detoxification**					
AL538_RS24635	Nitrite reductase large subunit	1.472	1.227	1.433	1
**DNA damage, repair and synthesis**					
AL538_RS18185	Deoxyribodipyrimidine photolyase	1.446	1.734	1.280	5
AL538_RS23750	DNA mismatch repair protein MutT	1.004	0,776	1.593	1
**Iron uptake, storage & utilization**					
AL538_RS26680	Ferric reductase	0.938	1.185	1.140	5
**Iron uptake/transport**					
AL538_RS21155	Iron permease	0.959	1.295	1.249	1
AL538_RS23515	Iron ABC transporter	1.203	1.009	0.937	1
**Iron storage**					
AL538_RS07875	Bacterioferritin	−2.859	−2.868	−3.496	2
AL538_RS12375	Ferredoxin, 2Fe-2S type, ISC system	−0.365	−0.256	−1.152	4
**Iron cluster biogenesis**					
AL538_RS16405	Cysteine desulfurase	−2.740	−3.362	−3.312	2
**Virulence factors involved in** ***V. harveyi*** **pathogenicity**					
AL538_RS26540	Putative heme utilization radical SAM enzyme HutW	1.028	1.301	0.458	5
AL538_RS20930	virK protein	0.708	1.912	1.755	5
**Quorum Sensing**					
AL538_RS06465	LuxR family transcriptional regulator	−1.543	−2.549	−2.843	3
AL538_RS19310	Sensor histidine kinase	1.253	1.080	1.148	1
AL538_RS25660	TonB-dependent siderophore receptor	1.338	1.520	1.380	5
**Chemotaxis, Motility & Biofilm**					
AL538_RS25425	Chemotaxis protein	0.892	1.340	1.235	5
AL538_RS21340	Flagellar biosynthesis protein FlhB	0.891	1.557	1.256	5
AL538_RS25995	Pilus assembly protein CpaF	1.152	1.522	1.409	5
**Secretion systems**					
AL538_RS15160	EscJ/YscJ/HrcJ family type III secretion inner membrane ring protein (T3SS)	1.077	1.212	1.477	1
AL538_RS23415	Type VI secretion protein	1.769	1.838	1.741	5
**Drug efflux & Antibiotic resistance**					
AL538_RS20560	Bcr/CflA family drug resistance efflux transporter	0.695	1.286	1.148	5
AL538_RS25145	Polyketide cyclase	0.622	1.104	1.046	5

## Discussion

Ongoing GW profoundly affects the physicochemical parameters and biological variety of natural aquatic systems. The relatively low level of dissolved organic matter in marine environments was shown to constrain bacterial growth in seawater microcosms (e.g^31,46–48^.) and is believed to be one of the major factors accountable for the presence of coccoid-like bacterioplankton in marine ecosystems^47,49^. To investigate the combined impact of elevated temperatures and limitation of carbon on *Vibrio* species, we used *V*. *harveyi* as a model organism and studied its persistence in seawater microcosms at 30 °C known to be within the upper limit of sea surface temperature observed in some areas of the global oceans^50^.

Analysis of cell morphology by fluorescence and electron microscopy revealed a discernible reduction of cell size by 3 day after exposure to microcosm conditions. *In silico* processing of cell images corroborated the overall reduction of cell size until the sixth day of incubation with no significant changes afterwards (Figs 2 and 3). The transition to the coccoid-like phenotype is consistent with the previously documented response of *V*. *harveyi* to carbon limitation^32^. Similar changes in morphology (i.e. size reduction and shape alterations) have been reported in old cell cultures of *V*. *parahaemolyticus* and *V*. *angustum* subjected to carbon starvation in marine mineral medium under laboratory conditions^51–53^. In natural aquatic systems, carbon concentration varies and brief periods of carbon abundance are normally interspersed with long periods of its scarcity. Therefore, it is quite common that free-living bacteria have reduced size in their natural habitats due to the shortage of carbon sources^54^. Comparison of three independent experiments performed with the same *V*. *harveyi* strain and under the same laboratory settings at 4 °C^32^, 20 °C^31^ and 30 °C (present work), demonstrates that reduction of cell size and occasional acquisition of a coccoid-like phenotype occur in a wide range of temperatures (i.e. from 4 °C to 30 °C). Moreover, elevated temperature (i.e. 30 °C) and shortage of carbon appear to limit the cell’s capacity to cope with stress and ultimately lead to the appearance of damaged cells seen in 21-day-old populations (Fig. 3). The appearance of damaged cells is congruent with a higher percentage of degraded RNA detected in *V*. *harveyi* cells after 14 and 21 days of incubation (data not shown). Moreover, an increase in the number of damaged cells is also consistent with a 90% loss of culturability within the entire population (Fig. 1) observed during the same period of time (i.e. after 14 days). Thus, in contrast to *V*. *harveyi* persistence and preservation of culturability at 20 °C^31^, its exposure to limitation of carbon at 30 °C leads to the higher vulnerability of the stressed cells to damage, which apparently causes their faster injury and potential death.

To learn about the major adaptation mechanisms that *V*. *harveyi* and likely other *Vibrio* species trigger to cope with elevated temperatures and limitation of nutrients in their natural habitats, we used RNA sequencing to obtain transcriptome profiles of *V*. *harveyi* following its incubation in seawater microcosm at 30 °C for 12 hours, 3 and 6 days. Comparison of these transcriptome data with those obtained for the control samples (initial inoculate) revealed a large number of genes that were differentially expressed during the incubation. Time series clustering analysis of the expression data (see Experimental procedures) revealed six distinct co-expressed clusters (Fig. 4), each comprising a set of genes with similar time-dependent expression profiles. The differentially expressed genes were further grouped according to their biological functions and presented in Table S1.

The fast morphological changes and accumulation of cells with damaged membranes (see above) indicate that *V*. *harveyi* was unable to properly control expression of genes involved in cell envelope biogenesis and associated metabolic pathways. The expression of phage shock proteins PspA, PspB and PspC (cluster 4) (Table 1), known for their role in relieving membrane stress^55^, is consistent with this explanation. In contrast to the previously described gradual upregulation of these genes in *V*. *harveyi* grown at 20 °C^31^, in this work, expression of these genes under limitation of carbon at 30 °C decreases after 6 day of incubation. This indicates the failure of *V*. *harveyi* to engage the products of the phage shock operon in the adaptation process.

Another example of “unexpected” response concerns lipid biogenesis and transport. Although the shrinkage of *V*. *harveyi* cell envelope associated with the reduction of cell size is anticipated to lead to a discharge of extra lipids and other membrane components for further recycling, we surprisingly found that expression of genes controlling lipid turnover decreased. A number of genes that are involved in lipid degradation via β-oxidation pathway (i.e. gene encoding phosphoglycerate kinase, glycerophosphoryl diester phosphodiesterase, acyl-CoA dehydrogenase, enoyl-CoA hydratase, beta-ketoacyl-ACP reductase, malonyl CoA-acyl carrier protein transacylase) (clusters 2 and 3) were strongly downregulated. The downregulation of genes controlling β-oxidation pathway as well as those involved in further conversion of acetyl-CoA, the key cellular metabolite generated via β-oxidation pathway, by the enzymes of the glyoxylate cycle (clusters 2 and 3) was unexpected and, in fact, was opposite to their upregulation at 20 °C observed in our previous work^31^. Thus, it seems likely, that, despite the considerably fast shrinkage of cell envelope and concomitant release of free lipids under carbon limiting conditions at 30 °C, *V*. *harveyi* fails to upregulate the key genes, the products of which are involved in lipid recycling. Nevertheless, the upregulation of some genes coding for long-chain fatty acid transporters (AL538_RS25015) could indicate that *V*. *harveyi* might try to deal with an excess of lipids accumulated due to reduction of cell size by changing their subcellular localization or exporting them outside of cells. In addition to some anticipated problems with lipid recycling, the shrinkage of cell envelope could additionally be hindered by discoordinate expression of major enzymes (for example, murein L,D-transpeptidase (cluster 4) and D-alanyl-D-alanine carboxypeptidase (cluster 3)) involved in peptidoglycan remodeling. Besides alterations in expression of genes controlling cell envelope biogenesis, another large group of the affected genes included those related to the *V*. *harveyi* central carbon metabolism. Analysis of their regulation revealed a fast downregulation of many genes involved in carboxylic acid metabolism (e.g. aconitate hydratase B (*acnB*), phosphoenolpyruvate carboxykinase; see Table S1 in Supplementary Data) as well as those controlling carbon storage such as the one encoding CsrB known for its roles in post-transcriptional control of gene expression. The downregulation of these genes is likely caused by limitation of carbon in seawater and it was observed already after 12 hours of incubation. Moreover, the initial rate of downregulation was considerably faster in the present study (i.e. at 30 °C) than that previously observed at 20 °C^31^, thus pointing to the overall increase in the rate of the cellular response to carbon scarcity at elevated temperatures. In addition, we found that, while decreasing the intensity of the central carbon metabolism, *V*. *harveyi* apparently attempts to switch to utilization of alternative carbon sources by upregulating a number of genes (e.g. alpha-amylase (cluster 1) and several alpha- and beta-mannosidase genes (belonging to clusters 1 and 5) encoding various hydrolases able to convert some polysaccharides naturally present in seawater into smaller metabolizable sugars.

An additional option to scavenge carbon sources from a carbon-limiting environments consists in secretion of proteinases able to degrade polypeptides potentially present in the environment (e.g. within the infected host cells^56^) into oligopeptides or amino acids for their further uptake by the starved cells. This strategy is less costly then *de novo* protein synthesis and is likely preferred by *V*. *harveyi* unable to maintain the required level of amino acids during its persistence in seawater microcosm under carbon-limiting conditions. Consistent with this scenario, the key enzymes involved in amino acid biosynthesis (i.e. arginine-succinate synthase, hydroxyproline-2-epimerase or ornithine cyclodeaminase from cluster 2; carbamoyl-phosphate synthase small subunit, cluster 3; glutamate synthase, cluster 6) were downregulated. Concomitantly, *V*. *harveyi* upregulated a number of genes (cluster 1) coding for various proteinases such as the subtilisin-serine peptidase S8 and protease M20. Most members of the peptidase S8 family are secreted and they are known to be implicated in the pathogenesis along with other metalloproteases by damaging host tissues^57,58^. The periplasmic peptidases belonging to M20 family are carboxy-metallo-proteases^59^ appear to assist the uptake of peptides by converting them into free amino acids in the periplasm. In contrast to the above examples, several protease genes were found to be downregulated. Their repression could be caused by different factors including the high cost of their operation (such as ATP-dependence of oligopeptidase A), reduced cellular needs in some specialized enzymes (e.g. proteases (HtpX and metalloprotease RseP) involved in protein quality control) or simply due to their redundancy (see Table S1).

Under carbon limitation and elevated temperature, *V*. *harveyi* also up- and downregulates a large number of transporter-encoding genes primarily found in clusters 1 and 5 (Fig. 4). Some upregulated genes control (i) the energy-dependent transport mediated by the TonB-ExbB-ExbD complex, (ii) multidrug efflux transporters that belong to the multidrug and toxic compound extrusion (MATE) family and (iii) membrane transport proteins of the major facilitator superfamily (MFS) that assist movement of small solutes across cell membranes. Many genes coding for ABC- and PTS-type transporters (clusters 2, 3 and 5) were downregulated, thus indicating that the transport of the corresponding molecules (certain sugars and amino acids) is likely taken over by alternative transport systems or is deactivated due to its redundancy.

Marine bacteria in the open ocean experience limitation of iron^60^, an essential metal vital for all living organisms. Consistent with the necessity to deal with low concentrations of iron in seawater microcosms, we observed that a number of genes involved in iron uptake/transport and storage were up- and downregulated, respectively. Moreover, incubation in seawater seems to activate some adaptive mechanisms aimed at protecting *V*. *harveyi* cells from the possible damage potentially caused by reactive oxygen species (ROS) and/or nitric oxide (NO). Consistently, we found that *V*. *harveyi* upregulated several stress-responsive genes (cluster 5) encoding catalase, alkyl hydroperoxide reductase subunit F, glutaredoxin as well as some nitrate reductases and cytochrome *c* nitrite reductase subunit NrfD able to minimize (or reduce) the damaging effects of ROS and / or NO.

Incubation at elevated temperature also affected regulation of *V*. *harveyi* genes controlling its virulence and ancillary mechanisms (i.e. production and secretion of virulence factors, biofilm formation and motility). We found that, in addition to increased expression of lytic enzymes including various glycosidases and proteinases, *V*. *harveyi* upregulated a number of genes encoding the Type III^61^, Type I^62^, Type IV^63^ and Type VI^64^ secretion systems involved in transport of virulence factors.

Similarly, upregulation was observed for structural proteins of flagella and pilus (flagellar biosynthesis proteins FlhB and FliR from cluster 5; pilus assembly proteins CpaB, CpaF, PapD, PilN, PilZ, TadB and TadE FliR from cluster 1 and 5) as well as ancillary enzymes such as peptidase A24 (gene AL538_RS01150) required for the type IV pilus formation, secretion of toxin and enzymes, gene transfer and biofilm formation^65^. The upregulation of the above genes can play an important role in cell motility and attachment during the initials steps of infection. Moreover, the overall increase in motility and capacity to attach to biotic surfaces could also increase *V*. *harveyi* chances to find new sources of food (e.g. *via* attachment to sea shells whose components (e.g. amino polysaccharides) can further be processed by secreted hydrolyzes to yield amino sugars in carbon-limiting environments.

Finally, we found that the gene coding for LuxR, a transcription factor mediating the quorum sensing (QS) response in a large variety of *Vibrio* spp^66^. was markedly downregulated, thereby suggesting that QS might not play a significant role in *V*. *harveyi* response to elevated SST and carbon limitation. Meanwhile, many genes encoding for proteins of two-component (sensor histidine kinase/response regulator) systems (CreC, UhpB and others, cluster 1 and 5) playing essential roles in sensing environmental signals and accordingly adjusting gene expression^67^ were upregulated. Several two-component systems previously described in vibrios and shown to be upregulated in the present study are involved in pathogenicity^68–70^. Therefore, their upregulation might enhance the pathogenic potential of *V*. *harveyi* exposed to elevated temperature and carbon limitation in seawater microcosm.

In summary, we observed a significant size reduction and acquisition of coccoid-like morphology by *V*. *harveyi* cells in seawater microcosms during their long-term incubation under carbon limitation at 30 °C. Since morphological changes, loss of culturability and cell damage are more pronounced at 30 °C than at 20 °C^31^, our data suggest that shortage of carbon at elevated temperature has a negative effect on *V*. *harveyi*. Despite its adverse effect on *V*. *harveyi* survival due to counter adaptive downregulation of some metabolic pathways (e.g. those controlling β-oxidation pathway), incubation in sea water microcosm at 30 °C readily enhances expression of many known virulence factors (i.e. lytic enzymes, components of the T3SS secretion system and iron-chelating compounds; reviewed in^71^) apparently accountable for the increased presence of pathogenic *Vibrio* species in the microbiota of marine invertebrate during the warm seasons^72^ and therefore potentially important for the spread of *Vibrio*-associated diseases in response to GW^9–12^. As the morphological, physiological and gene expression changes were observed in seawater microcosms, the rate and extent of *Vibrio* spp. responses to elevated temperature and limitation of carbon in their natural habitats are not necessarily the same and can likely be modulated by protozoan grazing, intensity of solar radiation and other environmental factors influencing the dynamics of marine ecosystems. Therefore, it is conceivable that some of the conclusions are limited by the experimental design.

## Electronic supplementary material

Table S1
