# Supplementary material for: Analysis of Vibrio harveyi adaptation in sea water microcosms at elevated temperature provides insights into the putative mechanisms of its persistence and spread in the time of global warming
Source: Sci Rep. 2019 Jan 22;9:289. doi: 10.1038/s41598-018-36483-0 (PMC6343004; doi:10.1038/s41598-018-36483-0)
Supplement: Supplementary file 1 — Table S1 [file 41598_2018_36483_MOESM1_ESM.docx]

**Analysis of *Vibrio harveyi* adaptation in sea water microcosms at elevated temperature provides insights into the putative mechanisms of its persistence and spread in the time of global warming**

**Itxaso Montánchez ^1^, Elixabet Ogayar^1^, Ander Hernández Plágaro^1^, Anna Esteve-Codina^2,3^, Jèssica Gómez Garrido^2, 3^, Maite Orruño^1,4^, Inés Arana^1,4^, Vladimir R. Kaberdin^1,4,5,6^**

**Supplementary data**

**Table S1.** **Highly up- and downregulated genes grouped according to their putative functions *in vivo*.** The systematic names are given according to the annotation of the reference *Vibrio harveyi* ATCC 43516 genome (available at <ftp://ftp.ncbi.nlm.nih.gov/genomes/genbank/>; accession number: GCA_001558435.1)

| **Systematic name** | **Gene product**  (specific biological pathway) | **Log_2_ fold change**  (time after exposure to seawater) | | | **Gene expression cluster** |
| --- | --- | --- | --- | --- | --- |
|  |  |  |  |  |  |
|  |  | 12 h | 3 d | 6 d |  |
| **Cell envelope stress** | | | | | |
| **AL538_RS20835** | Heat-shock protein | -0.616 | -1.048 | -1.215 | 6 |
| **AL538_RS16135** | Heat-shock protein HslJ | -1.093 | -0.796 | -1.732 | 4 |
| **Phage-shock-protein response** | | | | | |
| **AL538_RS15985** | Phage shock protein PspA | -0.908 | 0.819 | -1.526 | 4 |
| **AL538_RS15990** | Envelope stress response membrane protein PspB | -0.760 | 0.304 | -1.244 | 4 |
| **AL538_RS15995** | Envelope stress response membrane protein PspC | -1.042 | -0.263 | -1.819 | 4 |
| **Cell wall metabolism** | | | | | |
| **AL538_RS12080** | Lytic murein transglycosylase | -0.639 | -0.791 | -1.009 | 6 |
| **AL538_RS07100** |  | 0.759 | 0.747 | 1.007 | 1 |
| **AL538_RS11095** | UDP-N-acetylmuramate:L-alanyl-gamma-D-glutamyl-meso-diaminopimelate ligase | -1.112 | -0.950 | -1.084 | 2 |
| **Lipid biogenesis** | | | | | |
| **AL538_RS16785** | Lipid A biosynthesis lauroyl acyltransferase | 0.664 | 0.993 | 1.203 | 1 |
| **AL538_RS25885** | Choline dehydrogenase | 1.176 | 1.370 | 0.993 | 5 |
| **AL538_RS03425** | Acyl-CoA thioesterase | 0.878 | 1.704 | 1.073 | 5 |
| **AL538_RS13865** | Long-chain-fatty-acid--CoA ligase | -0.837 | -1.115 | -1.038 | 2 |
| **AL538_RS26865** | Glycerophosphoryl diester phosphodiesterase | -2.629 | -3.534 | -2.947 | 2 |
| **AL538_RS23675** | Acyl-CoA dehydrogenase | -2.053 | -2.768 | -2.308 | 2 |
| **AL538_RS23680** | Enoyl-CoA hydratase | -2.034 | -2.947 | -2.470 | 2 |
| **AL538_RS23685** |  | -2.182 | -2.589 | -2.503 | 2 |
| **AL538_RS27060** | Beta-ketoacyl-ACP reductase | -1.347 | -2.564 | -2.983 | 3 |
| **AL538_RS03900** | Malonyl CoA-acyl carrier protein transacylase | -1.815 | -2.683 | -2.368 | 3 |
| **AL538_RS15600** | Phospholipase C | -1.253 | -1.882 | -1.093 | 2 |
| **AL538_RS15740** | Cyclopropane-fatty-acyl-phospholipid synthase | -0.985 | -1.285 | -1.411 | 3 |
| **AL538_RS14940** | Tol-pal system-associated acyl-CoA thioesterase | -1.008 | -1.088 | -1.538 | 6 |
| **AL538_RS14125** | FAD-dependent oxidoreductase | 0.347 | 0.746 | 1.139 | 1 |
| **AL538_RS20940** |  | 0.378 | 1.121 | 0.854 | 5 |
| **AL538_RS04910** | Acetyl-CoA C-acyltransferase FadI | -0.092 | -1.133 | -0.269 | 3 |
| **Acetyl-CoA-dependent metabolism** | | | | | |
| **AL538_RS08475** | Acetyl-CoA carboxylase biotin carboxyl carrier protein subunit | -1.682 | -2.790 | -2.670 | 6 |
| **AL538_RS08480** | Acetyl-CoA carboxylase biotin carboxylase subunit | -1.694 | -2.530 | -2.391 | 3 |
| **AL538_RS05395** | Acetyl-CoA carboxylase carboxyl transferase subunit alpha | -1.273 | -2.388 | -2.011 | 3 |
| **AL538_RS04825** | Acetyl-CoA carboxylase carboxyl transferase subunit beta | -1.208 | -2.079 | -2.351 | 3 |
| **AL538_RS14965** | Peptidoglycan-associated lipoprotein | -1.663 | -2.943 | -2.801 | 3 |
| **AL538_RS14110** | 3-oxoacyl-ACP synthase | 0.933 | 1.219 | 1.249 | 5 |
| **Ethanol biosynthesis / biodegradation** | | | | | |
| **AL538_RS04550** | Bifunctional acetaldehyde-CoA/alcohol dehydrogenase | -1.470 | -3.161 | -2.871 | 3 |
| **AL538_RS17730** | Alcohol dehydrogenase | 0.799 | 0.465 | 1.083 | 1 |
| **AL538_RS09760** | Alcohol dehydrogenase | -1.727 | -3.140 | -2.750 | 3 |
| **AL538_RS23985** | NADH-dependent alcohol dehydrogenase | -1.359 | -1.956 | -2.513 | 6 |
| **AL538_RS18755** | S-(hydroxymethyl)glutathione dehydrogenase/class III alcohol dehydrogenase | -1.552 | -2.100 | -1.968 | 2 |
| **AL538_RS08550** | Zinc-dependent alcohol dehydrogenase | 1.133 | 0.660 | 1.148 | 1 |
| **Acetate biosynthesis (ATP production)** | | | | | |
| **AL538_RS08465** | Acetyl-CoA synthetase | -1.601 | -2.312 | -2.177 | 3 |
| **Energy production** | | | | | |
| **AL538_RS25345** | Electron transfer flavoprotein subunit alpha | -1.977 | -2.101 | -2.265 | 2 |
| **AL538_RS27345** | NADH dehydrogenase | 1.037 | 0.665 | 0.830 | 1 |
| **AL538_RS22660** | Cytochrome c | -0.669 | -1.578 | -1.741 | 3 |
| **AL538_RS10145** | Cytochrome c | -0.548 | -0.823 | -1.085 | 6 |
| **AL538_RS18830** | Cytochrome c biogenesis protein | 0.750 | 1.228 | 0.600 | 5 |
| **AL538_RS03195** | Cytochrome c nitrite reductase subunit NrfD | 0.565 | 1.438 | 0.764 | 5 |
| **AL538_RS26195** | Cytochrome c oxidase assembly protein | -0.492 | -0.705 | -1.359 | 4 |
| **AL538_RS26200** | Cytochrome c oxidase subunit I | -0.842 | -1.153 | -1.471 | 6 |
| **AL538_RS00780** | Cytochrome c oxidase. cbb3-type subunit I | -1.295 | -1.551 | -2.156 | 6 |
| **AL538_RS24750** | Cytochrome C554 | -1.499 | -1.123 | -1.848 | 4 |
| **AL538_RS14920** | Cytochrome d terminal oxidase subunit 1 | -1.139 | -1.270 | -1.210 | 2 |
| **AL538_RS17915** | Cytochrome d ubiquinol oxidase subunit II | 0.911 | 1.224 | 1.292 | 5 |
| **AL538_RS14925** | Cytochrome d ubiquinol oxidase subunit II | -0.934 | -0.369 | -1.066 | 4 |
| **AL538_RS07615** | Cytochrome-c oxidase. cbb3-type subunit III | -1.112 | -1.702 | -2.363 | 6 |
| **AL538_RS07640** | Cytochrome-c oxidase. cbb3-type subunit III | -1.112 | -1.702 | -2.363 | 6 |
| **AL538_RS00765** | Cytochrome-c oxidase. cbb3-type subunit III | -1.764 | -2.531 | -2.308 | 2 |
| **AL538_RS07650** | Cytochrome-c oxidase. cbb3-type subunit III | -1.142 | -1.068 | -1.850 | 6 |
| **AL538_RS26785** | Cytochrome-c peroxidase | 1.040 | 1.270 | 1.267 | 5 |
| **ATP synthesis coupled proton transport** | | | | | |
| **AL538_RS09620** | F0F1 ATP synthase subunit A | -1.654 | -1.656 | -2.178 | 6 |
| **AL538_RS09605** | F0F1 ATP synthase subunit delta | -1.768 | -3.170 | -2.672 | 3 |
| **AL538_RS09595** | F0F1 ATP synthase subunit gamma | -1.825 | -3.548 | -2.778 | 3 |
| **AL538_RS09625** | F0F1 ATP synthase subunit I | -1.568 | -2.783 | -2.312 | 3 |
| **Fumarate reductase (complex II)** | | | | | |
| **AL538_RS08270** | Succinate dehydrogenase/fumarate reductase iron-sulfur subunit | -0.974 | -1.628 | -0.798 | 2 |
| **AL538_RS08265** | Fumarate reductase flavoprotein subunit | -1.862 | -2.041 | -1.584 | 2 |
| **AL538_RS08275** | Fumarate reductase subunit C | -1.183 | -1.221 | -2.146 | 6 |
| **AL538_RS08280** | Fumarate reductase subunit D | -1.508 | -2.045 | -1.847 | 2 |
| **AL538_RS00660** | Formate dehydrogenase | -0.490 | -0.967 | -1.359 | 6 |
| **AL538_RS00665** | Formate dehydrogenase | -0.359 | -1.587 | -1.551 | 6 |
| **Amino acid metabolism** | | | | | |
| **Amino acid recycling** | | | | | |
| **AL538_RS23670** | Methylmalonate-semialdehyde dehydrogenase (CoA acylating) | -2.325 | -3.377 | -2.733 | 2 |
| **Alanine degradation** | | | | | |
| **AL538_RS15645** | Alanine dehydrogenase | -2.877 | -2.910 | -3.289 | 2 |
| **AL538_RS21930** | Alanine--glyoxylate aminotransferase | -1.603 | -2.442 | -1.489 | 2 |
| **AL538_RS06680** | Alanine--tRNA ligase | -0.928 | -1.365 | -1.566 | 6 |
| **Arginine and proline metabolism** | | | | | |
| **AL538_RS20570** | Arginine decarboxylase | 0.980 | 1.229 | 1.257 | 5 |
| **AL538_RS08005** | Arginine N-succinyltransferase | -1.016 | -1.592 | -1.194 | 2 |
| **AL538_RS13825** | Arginine--tRNA ligase | -0.526 | -0.715 | -1.032 | 6 |
| **AL538_RS10110** | Twin-arginine translocase subunit TatB | -0.944 | -1.268 | -1.630 | 6 |
| **AL538_RS27070** | Twin-arginine translocation pathway signal protein | -0.986 | -1.267 | -1.232 | 2 |
| **AL538_RS05580** | Proline--tRNA ligase | -0.750 | -1.298 | -0.870 | 3 |
| **AL538_RS01805** | Hydroxyproline-2-epimerase | -2.242 | -2.887 | -2.763 | 2 |
| **AL538_RS19730** | Ornithine carbamoyltransferase | 1.065 | 0.873 | 1.132 | 1 |
| **AL538_RS01790** | Ornithine cyclodeaminase | -2.714 | -4.302 | -3.209 | 2 |
| **AL538_RS18480** | Ornithine decarboxylase SpeF | 0.812 | 1.325 | 0.968 | 5 |
| **AL538_RS11685** | Carbamoyl-phosphate synthase small subunit | -1.555 | -2.798 | -2.228 | 3 |
| **AL538_RS07820** | Argininosuccinate synthase | -2.225 | -2.890 | -2.258 | 2 |
| **AL538_RS20565** | Agmatinase | 0.640 | 1.324 | 1.106 | 5 |
| **AL538_RS07825** | Acetylglutamate kinase | -1.058 | -1.119 | -1.333 | 2 |
| **Aspartate metabolism** | | | | | |
| **AL538_RS07290** | Aspartate carbamoyltransferase | -1.401 | -1.345 | -1.897 | 6 |
| **AL538_RS07295** | Aspartate carbamoyltransferase regulatory subunit | -1.251 | -1.976 | -1.289 | 2 |
| **AL538_RS19820** | Aspartate aminotransferase | 0.593 | 1.274 | 1.104 | 5 |
| **AL538_RS12535** | Aspartate aminotransferase family protein | -0.366 | -1.016 | -0.330 | 3 |
| **AL538_RS08010** | Aspartate aminotransferase family protein | -1.598 | -1.871 | -1.686 | 2 |
| **AL538_RS04840** | Aspartate-semialdehyde dehydrogenase | -0.658 | -1.609 | -1.441 | 3 |
| **AL538_RS04565** | Aspartate-semialdehyde dehydrogenase | -1.499 | -1.302 | -1.248 | 2 |
| **AL538_RS14895** | Aspartate--tRNA ligase | -1.120 | -0.774 | -1.138 | 2 |
| **Glutamate metabolism** | | | | | |
| **AL538_RS25000** | Glutamate synthase | -0.858 | -1.233 | -1.597 | 6 |
| **AL538_RS11745** | Glutamate synthase subunit beta | -1.595 | -2.359 | -1.828 | 2 |
| **AL538_RS13365** | Glutamate--tRNA ligase | -0.758 | -0.936 | -1.068 | 2 |
| **AL538_RS19575** | 1-pyrroline-5-carboxylate dehydrogenase | -0.487 | -0.696 | -1.220 | 6 |
| **Serine biosynthesis/degradation** | | | | | |
| **AL538_RS16375** | D-serine transporter DsdX | 0.814 | 1.221 | 0.964 | 5 |
| **AL538_RS16340** | 3-phosphoserine/phosphohydroxythreonine aminotransferase | -1.012 | -1.479 | -0.766 | 2 |
| **AL538_RS11815** | Homoserine kinase | -0.937 | -1.165 | -1.003 | 2 |
| **AL538_RS20985** | L-serine ammonia-lyase | -1.086 | -1.335 | -1.044 | 2 |
| **AL538_RS03000** | L-serine ammonia-lyase | -1.173 | -1.149 | -0.798 | 2 |
| **AL538_RS06050** | Phosphoserine phosphatase | -0.716 | -1.015 | -0.727 | 2 |
| **AL538_RS14635** | Serine protein kinase PrkA | -1.385 | -1.333 | -2.215 | 6 |
| **AL538_RS09945** | Serine/threonine protein kinase | 0.869 | 1.140 | 1.459 | 1 |
| **AL538_RS15670** | Serine--tRNA ligase | -1.094 | -1.263 | -1.430 | 2 |
| **AL538_RS06895** | D-3-phosphoglycerate dehydrogenase | -1.182 | -1.327 | -1.111 | 2 |
| **Valine. leucine and isoleucine biosynthesis / degradation** | | | | | |
| **AL538_RS09830** | Ketol-acid reductoisomerase | -0.120 | -1.001 | -0.870 | 3 |
| **Breakdown of tyrosine. phenylalanine and tryptophan** | | | | | |
| **AL538_RS01665** | 4-hydroxyphenylpyruvate dioxygenase | -2.624 | -2.794 | -3.415 | 2 |
| **AL538_RS01660** | Homogentisate 1.2-dioxygenase | -2.467 | -2.629 | -3.241 | 2 |
| **AL538_RS01650** | Maleylacetoacetate isomerase | -2.354 | -1.922 | -2.926 | 6 |
| **Stringent response** | | | | | |
| **AL538_RS11565** | Stringent starvation protein B | -1.121 | -0.789 | -1.168 | 2 |
| **Purine /pyrimidine biosynthesis** | | | | | |
| **AL538_RS25720** | Ribokinase | 0.859 | 0.909 | 1.231 | 1 |
| **AL538_RS15875** | Nucleoside-diphosphate sugar epimerase | -1.163 | -1.580 | -1.245 | 2 |
| **AL538_RS06065** | Phosphopentomutase | -1.607 | -2.371 | -2.171 | 3 |
| **AL538_RS03240** | Ribonucleoside-diphosphate reductase subunit alpha | -1.121 | -1.960 | -2.065 | 3 |
| **AL538_RS03245** | Ribonucleotide-diphosphate reductase subunit beta | -1.553 | -2.794 | -2.503 | 3 |
| **AL538_RS14510** | Uridine phosphorylase | -1.682 | -2.946 | -3.119 | 3 |
| **AL538_RS07295** | Aspartate carbamoyltransferase regulatory subunit | -1.251 | -1.976 | -1.289 | 2 |
| **AL538_RS22215** | Dihydroorotase | -0.689 | -0.495 | -1.008 | 4 |
| **Nucleotide salvage pathways (nucleotide recycling)** | | | | | |
| **AL538_RS13630** | Adenylate kinase | -1.563 | -2.207 | -2.102 | 3 |
| **Carbon metabolism** | | | | | |
| **Citrate. glyoxylate & 2-methylcitrate cycles** | | | | | |
| **AL538_RS17025** | Citrate synthase/methylcitrate synthase | -2.020 | -1.656 | -2.315 | 6 |
| **AL538_RS13760** | 2-oxoglutarate dehydrogenase subunit E1 | -1.543 | -1.706 | -1.613 | 2 |
| **AL538_RS13765** | Dihydrolipoamide succinyltransferase | -1.233 | -1.028 | -1.384 | 2 |
| **AL538_RS12280** | Isocitrate lyase | -1.318 | -1.596 | -1.452 | 2 |
| **AL538_RS06380** | Aconitate hydratase B | -1.588 | -2.634 | -2.372 | 3 |
| **AL538_RS17035** | 2-methylaconitate cis-trans isomerase PrpF | -1.876 | -1.908 | -1.961 | 2 |
| **AL538_RS10235** | Phosphoenolpyruvate carboxykinase (ATP) | -1.458 | -3.015 | -2.504 | 3 |
| **AL538_RS11240** | 2-isopropylmalate synthase [Vibrio] | -1.441 | -1.289 | -1.370 |  |
| **AL538_RS07840** | Phosphoenolpyruvate carboxylase | -0.996 | -1.292 | -1.183 | 2 |
| **Glycolysis and gluconeogenesis** | | | | | |
| **AL538_RS06930** | Phosphoglycerate kinase | -1.341 | -2.545 | -2.503 | 3 |
| **AL538_RS10235** | Phosphoenolpyruvate carboxykinase (ATP) | -1.458 | -3.015 | -2.504 | 3 |
| **AL538_RS04660** | Type I glyceraldehyde-3-phosphate dehydrogenase | -1.325 | -2.871 | -2.660 | 3 |
| **AL538_RS01195** | Glucose-6-phosphate dehydrogenase | -0.672 | -0.609 | -1.126 | 6 |
| **AL538_RS20635** | 6-phospho-beta-glucosidase | -0.557 | -0.777 | -1.112 | 6 |
| **Pyruvate-dependent pathways** | | | | | |
| **AL538_RS14160** | Isochorismatase | 0.839 | 1.241 | 1.159 | 5 |
| **AL538_RS14780** | 3-phosphoshikimate 1-carboxyvinyltransferase | -1.126 | -1.715 | -1.390 | 3 |
| **Protein turnover and folding** | | | | | |
| **Protein degradation** | | | | | |
| **AL538_RS12295** | Serine protease | 0.744 | 1.079 | 1.240 | 1 |
| **AL538_RS01370** | Alkaline serine protease | -1.378 | -3.486 | -2.372 | 4 |
| **AL538_RS11515** | Serine endoprotease DegQ | -0.449 | -1.373 | -1.586 | 3 |
| **AL538_RS07400** | Metalloprotease PmbA | -0.849 | -0.801 | -1.178 | 6 |
| **AL538_RS06130** | Protease | 1.034 | 1.137 | 1.308 | 1 |
| **AL538_RS15715** | Protease HtpX | -0.821 | -0.978 | -1.552 | 6 |
| **AL538_RS05440** | RIP metalloprotease RseP | -0.197 | -1.289 | -1.086 | 3 |
| **AL538_RS20315** | Zn-dependent protease | -1.482 | -1.687 | -2.175 | 6 |
| **AL538_RS12810** | Aminoacyl-histidine dipeptidase | -1.960 | -2.991 | -2.215 | 2 |
| **AL538_RS10020** | Oligopeptidase A | -1.105 | -1.895 | -2.198 | 6 |
| **AL538_RS01615** | Peptidase | -0.896 | -1.139 | -1.123 | 2 |
| **AL538_RS20505** | Peptidase | 1.067 | 1.355 | 1.071 | 5 |
| **AL538_RS18730** | Peptidase | -0.014 | -1.023 | -0.910 | 3 |
| **AL538_RS08240** | Peptidase | -0.446 | -1.010 | -0.269 | 3 |
| **AL538_RS01150** | Peptidase A24 | 0.249 | 1.061 | 1.581 | 1 |
| **AL538_RS05230** | Peptidase M15 | -0.419 | -0.572 | -1.261 | 4 |
| **AL538_RS25460** | Peptidase M19 | 0.957 | 0.900 | 1.036 | 1 |
| **AL538_RS01675** | Peptidase M20 | 0.859 | 0.886 | 1.368 | 1 |
| **AL538_RS24140** | Peptidase M50 | 1.118 | 0.888 | 1.354 | 1 |
| **AL538_RS00775** | Peptidase S41 | -1.639 | -2.045 | -1.936 | 2 |
| **AL538_RS25255** | Peptidase S8 | 0.698 | 0.813 | 1.005 | 1 |
| **AL538_RS11905** | Peptide chain release factor 1 | -0.366 | -1.288 | -0.954 | 3 |
| **AL538_RS12385** | Aminopeptidase PepB | -1.212 | -1.429 | -0.934 | 2 |
| **AL538_RS07245** | Cytosol aminopeptidase | -0.912 | -1.460 | -1.109 | 3 |
| **AL538_RS18135** | D-alanyl-D-alanine carboxypeptidase | -1.318 | -2.530 | -2.336 | 3 |
| **AL538_RS14260** | Endopeptidase La | -0.946 | -1.745 | -1.151 | 3 |
| **AL538_RS03145** | Murein L.D-transpeptidase | -0.373 | 0.473 | -1.237 | 4 |
| **AL538_RS01935** | Oligoendopeptidase F | -1.118 | -1.260 | -1.409 | 2 |
| **AL538_RS17705** | Xaa-His dipeptidase | 1.352 | 1.540 | 1.276 | 5 |
| **AL538_RS01705** | Xaa-Pro aminopeptidase | -2.220 | -4.042 | -3.019 | 3 |
| **AL538_RS09315** | Xaa-Pro aminopeptidase | -1.018 | -1.271 | -1.375 | 2 |
| **AL538_RS24480** | Xaa-Pro dipeptidase | -1.700 | -2.530 | -2.663 | 3 |
| **Protein folding** | | | | | |
| **AL538_RS25315** | Molecular chaperone | 0.594 | 1.004 | 1.011 | 5 |
| **AL538_RS05430** | Molecular chaperone | -1.777 | -1.784 | -2.056 | 2 |
| **AL538_RS12735** | Molecular chaperone DnaJ | -0.636 | -1.307 | -0.720 | 3 |
| **AL538_RS10240** | Molecular chaperone Hsp33 | -0.578 | -1.187 | -1.147 | 3 |
| **AL538_RS00770** | CcoQ/FixQ family Cbb3-type cytochrome c oxidase assembly chaperone | -1.696 | -1.968 | -2.171 | 6 |
| **AL538_RS16020** | Lipase chaperone | 1.174 | 0.964 | 1.124 | 1 |
| **AL538_RS00890** | RNA chaperone ProQ | -1.198 | -1.487 | -2.016 | 6 |
| **AL538_RS12125** | ATP-dependent chaperone ClpB | -1.046 | -1.499 | -1.898 | 6 |
| **Antioxidative defence** | | | | | |
| **AL538_RS19980** | Alkyl hydroperoxide reductase subunit F | 0.443 | 0.980 | 1.170 | 5 |
| **AL538_RS17640** | Catalase | 1.260 | 2.075 | 1.668 | 5 |
| **AL538_RS15575** | Catalase-peroxidase | -1.254 | -3.259 | -2.811 | 3 |
| **AL538_RS05275** | Arsenate reductase (glutaredoxin) | -1.339 | -0.582 | -1.802 | 4 |
| **AL538_RS21845** | Glutaredoxin. GrxB family | 0.713 | 1.418 | 0.700 | 5 |
| **AL538_RS04530** | Monothiol glutaredoxin. Grx4 family | -0.871 | -0.911 | -1.563 | 6 |
| **Nitric oxide detoxification** | | | | | |
| **AL538_RS03195** | Cytochrome c nitrite reductase subunit NrfD | 0.565 | 1.438 | 0.764 | 5 |
| **AL538_RS22675** | Nitrate reductase | -0.473 | -0.429 | -1.369 | 4 |
| **AL538_RS25185** |  | 0.673 | 1.166 | 0.939 | 5 |
| **AL538_RS25225** |  | 0.607 | 0.929 | 1.040 | 5 |
| **AL538_RS24635** | Nitrite reductase large subunit | 1.472 | 1.227 | 1.433 | 1 |
| **AL538_RS13585** | Nitrogen regulatory protein P-II | -1.303 | -1.864 | -1.960 | 6 |
| **AL538_RS05380** | Nitrogen regulatory protein P-II | -1.132 | -1.471 | -1.631 | 6 |
| **Sigma E – ChrR system** | | | | | |
| **AL538_RS06820** | Anti-sigma E factor | -0.557 | -0.095 | -1.517 | 4 |
| **AL538_RS06810** | Positive regulator of sigma E activity | -0.326 | -0.313 | -1.179 | 4 |
| **DNA damage. repair and synthesis** | | | | | |
| **AL538_RS13710** | Replication initiation regulator SeqA | -1.042 | -1.042 | -1.632 | 6 |
| **AL538_RS18185** | Deoxyribodipyrimidine photolyase | 1.446 | 1.734 | 1.280 | 5 |
| **AL538_RS10985** | ATP-dependent RNA helicase | -0.826 | -1.149 | -0.665 | 2 |
| **AL538_RS19845** | DEAD/DEAH box helicase | 0.542 | 1.216 | 1.155 | 5 |
| **AL538_RS14190** | RNA helicase | 0.815 | -0.026 | 1.028 | 1 |
| **AL538_RS06915** | DNA-binding protein | -2.431 | -2.659 | -2.544 | 2 |
| **AL538_RS15785** |  | -1.204 | -1.867 | -1.031 | 2 |
| **AL538_RS26130** |  | 1.140 | 1.660 | 1.295 | 1 |
| **AL538_RS07560** | Single-stranded DNA-binding protein | -1.535 | -1.806 | -1.838 | 2 |
| **AL538_RS23750** | DNA mismatch repair protein MutT | 1.004 | 0.776 | 1.593 | 1 |
| **AL538_RS09695** | DNA polymerase III subunit beta | -1.011 | -1.309 | -1.308 | 2 |
| **AL538_RS14655** | Exonuclease | 0.843 | 1.358 | 1.059 | 5 |
| **AL538_RS03225** | DNA gyrase subunit A | -0.368 | -0.454 | -1.216 | 4 |
| **Transport** | | | | | |
| **Energy-dependent transport TonB-ExbB-ExbD complex** | | | | | |
| **AL538_RS26550** | Biopolymer transporter ExbB | 1.623 | 2.108 | 1.740 | 5 |
| **AL538_RS19445** | TonB-system energizer ExbB | 0.201 | 1.186 | 1.025 | 5 |
| **ABC transporters** | | | | | |
| **AL538_RS01240** | ABC transporter | 0.153 | 1.407 | 0.028 | 5 |
| **AL538_RS25410** |  | -2.188 | -1.852 | -1.608 | 2 |
| **AL538_RS27075** |  | 0.982 | 0.893 | 1.015 | 1 |
| **AL538_RS17835** |  | 0.662 | 0.415 | 1.057 | 1 |
| **AL538_RS06445** |  | 0.775 | 0.867 | 1.108 | 1 |
| **AL538_RS01700** | ABC transporter ATP-binding protein | -2.419 | -3.833 | -2.944 | 3 |
| **AL538_RS00900** |  | -1.257 | -1.754 | -1.936 | 3 |
| **AL538_RS18590** |  | 0.725 | 1.253 | 1.153 | 5 |
| **AL538_RS09900** |  | -1.267 | -2.130 | -2.044 | 3 |
| **AL538_RS22125** |  | -1.420 | -1.759 | -1.554 | 2 |
| **AL538_RS00590** |  | -0.943 | -0.944 | -1.249 | 6 |
| **AL538_RS20535** |  | 1.253 | 1.520 | 0.914 | 5 |
| **AL538_RS06320** |  | 1.171 | 0.921 | 1.160 | 1 |
| **AL538_RS01775** | ABC transporter permease | -2.482 | -3.027 | -2.565 | 2 |
| **AL538_RS09915** |  | -0.508 | -1.138 | -0.746 | 3 |
| **AL538_RS25680** |  | 0.789 | 1.230 | 0.841 | 5 |
| **AL538_RS19325** |  | 1.212 | 1.748 | 1.369 | 5 |
| **AL538_RS20070** |  | 1.311 | 1.792 | 1.402 | 5 |
| **AL538_RS20295** |  | 1.509 | 2.157 | 1.920 | 5 |
| **AL538_RS09910** |  | -1.454 | -1.581 | -1.890 | 2 |
| **AL538_RS26870** | ABC transporter substrate-binding protein | -2.683 | -4.749 | -3.279 | 2 |
| **AL538_RS17590** |  | -0.742 | -1.337 | -1.215 | 3 |
| **AL538_RS10445** | ABC transporter substrate-binding protein | -0.466 | -1.105 | -0.901 | 2 |
| **AL538_RS22820** |  | 0.646 | 0.898 | 1.237 | 1 |
| **AL538_RS14680** |  | -1.806 | -2.848 | -2.324 | 3 |
| **AL538_RS17540** | ABC transporter substrate-binding protein | -0.222 | -0.550 | -1.112 | 6 |
| **AL538_RS19380** | Amino acid ABC transporter | -1.236 | -1.698 | -1.907 | 3 |
| **AL538_RS23960** | Amino acid ABC transporter permease | -0.887 | 0.650 | -1.101 | 4 |
| **AL538_RS00950** | Amino acid ABC transporter substrate-binding protein | -1.188 | -2.888 | -2.524 | 3 |
| **AL538_RS09685** |  | -1.417 | -1.807 | -2.100 | 6 |
| **AL538_RS23965** |  | -1.390 | -2.612 | -2.090 | 3 |
| **AL538_RS08400** | C4-dicarboxylate ABC transporter | -1.633 | -1.850 | -1.721 | 2 |
| **AL538_RS15465** |  | 0.267 | 1.105 | 0.605 | 5 |
| **AL538_RS05790** |  | 0.957 | 1.072 | 0.681 | 5 |
| **AL538_RS20665** | C4-dicarboxylate ABC transporter permease | 0.868 | 1.211 | 1.186 | 5 |
| **AL538_RS14220** |  | 0.884 | 1.272 | 0.951 | 5 |
| **AL538_RS11130** | C4-dicarboxylate ABC transporter substrate-binding protein | -1.327 | -2.314 | -2.223 | 3 |
| **AL538_RS25870** | Choline ABC transporter ATP-binding protein | 0.930 | 0.579 | 1.050 | 1 |
| **AL538_RS17400** | Cobalt ABC transporter permease | 0.847 | 0.781 | 1.114 | 1 |
| **AL538_RS17205** | Heme acquisition ABC transporter HasD | 0.602 | 0.754 | 1.029 | 1 |
| **AL538_RS26560** | Hemin ABC transporter substrate-binding protein | 1.333 | 0.179 | 0.996 | 1 |
| **AL538_RS14675** | Histidine/lysine/arginine/ornithine ABC transporter ATP-binding protein HisP | -1.671 | -1.556 | -1.824 | 2 |
| **AL538_RS14685** | Histidine/lysine/arginine/ornithine ABC transporter permease HisQ | -1.310 | -1.695 | -1.841 | 2 |
| **AL538_RS14605** | Lipid ABC transporter permease/ATP-binding protein | -0.666 | -0.898 | -1.634 | 6 |
| **AL538_RS03595** | Macrolide ABC transporter ATP-binding protein | 1.481 | 0.502 | 1.199 | 1 |
| **AL538_RS25190** | Nitrate ABC transporter ATP-binding protein | 1.037 | 0.927 | 1.429 | 1 |
| **AL538_RS01690** | Peptide ABC transporter permease | -1.649 | -2.492 | -2.032 | 3 |
| **AL538_RS04075** |  | -1.067 | -0.772 | -0.902 | 2 |
| **AL538_RS01680** | Peptide ABC transporter substrate-binding protein | -2.146 | -4.524 | -3.021 | 3 |
| **AL538_RS01780** | Polyamine ABC transporter ATP-binding protein | -2.676 | -3.963 | -3.018 | 2 |
| **AL538_RS01770** | Polyamine ABC transporter permease | -2.572 | -2.637 | -2.720 | 2 |
| **AL538_RS20900** | Putative 2-aminoethylphosphonate ABC transporter substrate-binding protein | -2.145 | -3.945 | -2.776 | 3 |
| **AL538_RS01795** | Spermidine/putrescine ABC transporter substrate-binding protein | -2.751 | -4.780 | -3.382 | 2 |
| **AL538_RS04140** | Excinuclease ABC subunit B | 0.672 | 0.777 | 1.042 | 1 |
| **Sec-dependent translocation** | | | | | |
| **AL538_RS08685** | Preprotein translocase subunit SecE | -0.640 | -1.550 | -1.241 | 3 |
| **AL538_RS14365** | Preprotein translocase subunit Tim44 | -0.922 | -1.678 | -1.686 | 3 |
| **Maltose / maltodextrines transport** | | | | | |
| **AL538_RS09720** | Alpha-amylase | 0.858 | 0.653 | 1.055 | 1 |
| **AL538_RS20165** | Maltose operon protein | -1.820 | -2.931 | -2.592 | 3 |
| **Phosphate transport (*pstSCAB operon*)** | | | | | |
| **AL538_RS17175** | Phosphate ABC transporter permease subunit PstC | 1.465 | 1.515 | 1.782 | 1 |
| **AL538_RS17280** |  | -1.142 | -1.560 | -1.800 | 6 |
| **AL538_RS25505** | Phosphate ABC transporter substrate-binding protein | -0.948 | -1.124 | -1.076 | 2 |
| **AL538_RS17285** | Phosphate ABC transporter substrate-binding protein | -2.034 | -4.274 | -3.377 | 3 |
| **AL538_RS17275** | Phosphate ABC transporter. permease protein PstA | -1.414 | -2.649 | -2.232 | 3 |
| **AL538_RS11445** | Phosphate transport regulator | -0.741 | -1.150 | -1.351 | 6 |
| **AL538_RS04700** | Alkaline phosphatase | -1.825 | -2.799 | -2.727 | 3 |
| **sn-Glycerol 3-phosphate transport system** | | | | | |
| **AL538_RS26875** | Sn-glycerol-3-phosphate transport system.-binding protein inner membrane component | -1.535 | -2.186 | -1.711 | 2 |
| **Arginine transport** | | | | | |
| **AL538_RS00965** | Arginine ABC transporter ATP-binding protein | -0.989 | -1.246 | -0.880 | 2 |
| **AL538_RS25420** | Arginine ABC transporter ATP-binding protein ArtP | -1.795 | -3.056 | -1.844 | 2 |
| **AL538_RS25415** | Arginine ABC transporter substrate-binding protein | -2.022 | -3.010 | -2.415 | 2 |
| **Other transporters** | | | | | |
| **AL538_RS19885** | Threonine transporter RhtB | 1.013 | 1.686 | 0.990 | 5 |
| **AL538_RS19740** | Amino acid permease | 0.523 | 1.022 | 1.190 | 5 |
| **AL538_RS06910** | Amino acid transporter | -2.510 | -3.611 | -2.367 | 2 |
| **AL538_RS13610** | Anion permease | -0.068 | 1.425 | -0.175 | 5 |
| **AL538_RS17480** | L-lactate permease | -1.622 | -1.498 | -1.687 | 2 |
| **AL538_RS12815** | NCS2 family permease | 0.674 | 1.177 | 0.826 | 5 |
| **AL538_RS24605** | Anaerobic C4-dicarboxylate transporter | 0.868 | 1.553 | 1.429 | 5 |
| **AL538_RS18075** | AI-2E family transporter | -1.038 | -1.552 | -1.368 | 3 |
| **AL538_RS25455** | BCCT transporter | 0.519 | 1.145 | 0.450 | 5 |
| **AL538_RS26905** | Cation transporter | 0.774 | 2.088 | 0.807 | 5 |
| **AL538_RS13110** | Cobalt transporter | -1.115 | -1.292 | -1.490 | 2 |
| **AL538_RS26660** | Copper transporter | 1.044 | 1.275 | 1.432 | 1 |
| **AL538_RS17580** | EamA family transporter | -1.354 | -0.854 | -1.822 | 4 |
| **AL538_RS17655** |  | 0.699 | 1.191 | 0.524 | 5 |
| **AL538_RS03560** |  | 0.335 | 0.381 | 1.063 | 1 |
| **AL538_RS10080** |  | 0.859 | 0.884 | 1.222 | 1 |
| **AL538_RS25595** |  | 0.723 | 1.235 | 1.678 | 1 |
| **AL538_RS01055** |  | 0.950 | 1.603 | 1.870 | 5 |
| **AL538_RS23630** |  | 0.589 | 0.843 | 1.046 | 1 |
| **AL538_RS16860** | Efflux transporter periplasmic adaptor subunit | 0.290 | 1.217 | 1.291 | 5 |
| **AL538_RS26665** |  | 1.095 | 1.036 | 1.424 | 1 |
| **AL538_RS23890** |  | 1.262 | 0.254 | 1.730 | 1 |
| **AL538_RS25350** | Electron transporter RnfB | -2.009 | -2.692 | -2.495 | 2 |
| **AL538_RS15895** | Formate transporter FocA | -1.227 | -2.626 | -2.400 | 3 |
| **AL538_RS24075** |  | 1.210 | 1.168 | 1.754 | 1 |
| **AL538_RS26880** | Glycerol-3-phosphate transporter | -1.884 | -1.757 | -1.192 | 2 |
| **AL538_RS26795** | Long-chain fatty acid transporter | 0.754 | 1.261 | 0.299 | 5 |
| **AL538_RS25015** |  | 0.640 | 0.909 | 1.212 | 1 |
| **AL538_RS17795** | Lysine transporter LysE | 0.978 | 0.909 | 1.002 | 1 |
| **AL538_RS20975** | Manganese transporter | 1.600 | 1.992 | 1.690 | 5 |
| **AL538_RS01420** | MATE family efflux transporter | 0.906 | 0.972 | 1.080 | 1 |
| **AL538_RS08350** |  | 1.061 | 0.762 | 1.096 | 1 |
| **AL538_RS24425** |  | 0.825 | 1.474 | 1.177 | 5 |
| **AL538_RS18805** |  | 1.097 | 1.564 | 1.286 | 5 |
| **AL538_RS19005** |  | 0.350 | 1.011 | 0.598 | 5 |
| **AL538_RS05180** | Melibiose:sodium transporter MelB | 0.816 | 0.661 | 1.085 | 1 |
| **AL538_RS23230** | MFS transporter | 1.150 | 1.416 | 1.364 | 1 |
| **AL538_RS01870** |  | -1.241 | -0.311 | -1.380 | 4 |
| **AL538_RS25050** |  | 0.720 | 1.015 | 1.083 | 1 |
| **AL538_RS01445** |  | 0.887 | 1.458 | 1.150 | 5 |
| **AL538_RS22405** |  | 0.689 | 1.119 | 1.177 | 5 |
| **AL538_RS18600** |  | 0.597 | 1.427 | 1.206 | 5 |
| **AL538_RS03545** |  | 1.197 | 1.520 | 2.155 | 1 |
| **AL538_RS26190** |  | -1.089 | -1.177 | -2.073 | 6 |
| **AL538_RS24565** |  | 0.497 | 1.197 | 0.699 | 5 |
| **AL538_RS17405** |  | 1.207 | 1.630 | 1.251 | 5 |
| **AL538_RS18425** |  | 0.914 | 1.609 | 1.494 | 5 |
| **AL538_RS21850** |  | 1.249 | 1.445 | 1.537 | 1 |
| **AL538_RS20680** | NADH:ubiquinone reductase (Na(+)-transporting) subunit B | 0.921 | 1.504 | 1.047 | 5 |
| **AL538_RS05660** | NADH:ubiquinone reductase (Na(+)-transporting) subunit C | -1.130 | -1.552 | -2.129 | 6 |
| **AL538_RS21195** |  | 0.334 | 1.104 | 0.678 | 5 |
| **AL538_RS05655** | NADH:ubiquinone reductase (Na(+)-transporting) subunit D | -0.517 | -0.993 | -1.483 | 6 |
| **AL538_RS05650** | NADH:ubiquinone reductase (Na(+)-transporting) subunit E | -1.335 | -1.664 | -2.340 | 6 |
| **AL538_RS05645** | NADH:ubiquinone reductase (Na(+)-transporting) subunit F | -1.208 | -1.823 | -1.941 | 3 |
| **AL538_RS25705** | Nicotinamide mononucleotide transporter | 0.583 | 1.267 | 0.486 | 5 |
| **AL538_RS06080** | NupC/NupG family nucleoside CNT transporter | -1.506 | -2.402 | -2.384 | 3 |
| **AL538_RS19095** | PTS ascorbate transporter subunit IIBC | 1.090 | 1.290 | 1.338 | 5 |
| **AL538_RS22020** | PTS fructose transporter subunit IIB | -0.993 | -0.833 | -1.364 | 6 |
| **AL538_RS22005** | PTS fructose transporter subunit IIC | 0.486 | 1.119 | 0.653 | 5 |
| **AL538_RS14995** | PTS fructose-like transporter subunit EIIC | 0.510 | 1.145 | 1.203 | 5 |
| **AL538_RS13505** | PTS glucose transporter subunit IIA | -1.322 | -2.533 | -2.634 | 3 |
| **AL538_RS14335** | PTS glucose transporter subunit IIB | 0.735 | 0.892 | 1.420 | 1 |
| **AL538_RS03855** | PTS glucose transporter subunit IIBC | -1.263 | -1.644 | -2.161 | 6 |
| **AL538_RS17670** | PTS mannitol transporter subunit IIA | 1.036 | 1.597 | 1.373 | 5 |
| **AL538_RS26355** | PTS mannitol transporter subunit IIBC | 1.188 | 1.438 | 1.043 | 5 |
| **AL538_RS17700** | PTS mannose transporter subunit IIC | 0.757 | 1.002 | 1.159 | 1 |
| **AL538_RS13670** | PTS N-acetylmuramic acid transporter subunit IIBC | -1.265 | -1.290 | -1.867 | 6 |
| **AL538_RS15325** | PTS sucrose transporter subunit IIBC | 1.044 | 1.311 | 1.225 | 5 |
| **AL538_RS02080** | RND transporter | 0.833 | 1.032 | 1.159 | 1 |
| **AL538_RS26620** | Sodium:proline symporter | 0.931 | 1.445 | 1.042 | 5 |
| **AL538_RS19580** |  | -1.148 | -1.084 | -1.286 | 2 |
| **AL538_RS16040** | Sodium-dependent transporter | -0.590 | -0.627 | -1.064 | 6 |
| **AL538_RS16130** | Sodium-independent anion transporter | 0.648 | 2.154 | -0.150 | 5 |
| **AL538_RS17905** | Transporter | 0.444 | 1.747 | 1.361 | 5 |
| **AL538_RS20000** |  | -0.927 | -1.686 | -1.117 | 3 |
| **AL538_RS24445** |  | 0.726 | 0.507 | 1.104 | 1 |
| **AL538_RS15560** |  | 0.707 | 1.240 | 1.270 | 5 |
| **AL538_RS21740** |  | 1.129 | 1.214 | 1.357 | 1 |
| **AL538_RS09840** |  | -0.707 | -0.943 | -1.187 | 6 |
| **AL538_RS15480** |  | 0.688 | 0.856 | 1.230 | 1 |
| **AL538_RS15315** | Porin | 1.228 | 1.243 | 1.429 | 1 |
| **Translation** | | | | | |
| **AL538_RS10220** | D-tyrosyl-tRNA(Tyr) deacylase | -1.153 | -1.650 | -0.910 | 2 |
| **AL538_RS06035** | Elongation factor G | -1.316 | -2.547 | -2.559 | 3 |
| **AL538_RS08295** | Elongation factor P | -0.416 | -0.686 | -1.560 | 4 |
| **AL538_RS03335** | Elongation factor P-like protein YeiP | -0.811 | -1.366 | -2.202 | 6 |
| **AL538_RS12075** | Energy-dependent translational throttle protein EttA | -1.550 | -2.247 | -2.244 | 3 |
| **AL538_RS06175** | Translation initiation factor IF-2 | -0.946 | -1.702 | -1.504 | 3 |
| **AL538_RS02125** | Translation initiation factor IF-3 | -1.436 | -2.093 | -2.476 | 6 |
| **AL538_RS10200** | Translational GTPase TypA | -0.795 | -1.083 | -1.677 | 6 |
| **tRNA and Ribosome biogenesis** | | | | | |
| **AL538_RS14735** | 23S rRNA pseudouridylate synthase | 0.435 | 0.471 | 1.027 | 1 |
| **AL538_RS16205** | 16S rRNA pseudouridine(516) synthase | 0.829 | 1.243 | 0.717 | 5 |
| **AL538_RS16200** | 16S rRNA pseudouridine(516) synthase | 1.080 | 0.921 | 1.034 | 1 |
| **Ribosomal proteins** | | | | | |
| **AL538_RS10940** | 30S ribosomal protein S11 | -0.742 | -1.618 | -1.978 | 6 |
| **AL538_RS07900** | 30S ribosomal protein S12 [Vibrionaceae] | -1.454 | -2.108 | -2.716 | 6 |
| **AL538_RS10935** | 30S ribosomal protein S13 | -1.239 | -1.082 | -1.742 | 6 |
| **AL538_RS10845** | 30S ribosomal protein S19 | -1.068 | -0.698 | -1.896 | 4 |
| **AL538_RS11980** | 30S ribosomal protein S20 | -1.096 | -0.304 | -1.460 | 4 |
| **AL538_RS10855** | 30S ribosomal protein S3 | -0.631 | -0.915 | -1.570 | 6 |
| **AL538_RS10945** | 30S ribosomal protein S4 | -0.871 | -1.974 | -2.224 | 3 |
| **AL538_RS20585** | 30S ribosomal protein S6--L-glutamate ligase | -0.896 | -1.369 | -1.560 | 6 |
| **AL538_RS07895** | 30S ribosomal protein S7 | -0.665 | -1.414 | -1.753 | 6 |
| **AL538_RS10895** | 30S ribosomal protein S8 | -0.477 | -0.576 | -1.438 | 4 |
| **AL538_RS11540** | 30S ribosomal protein S9 | -1.169 | -0.686 | -1.635 | 4 |
| **AL538_RS11535** | 50S ribosomal protein L13 | -0.915 | -0.815 | -1.966 | 4 |
| **AL538_RS10875** | 50S ribosomal protein L14 [Vibrionaceae] | -0.529 | -0.802 | -1.056 | 6 |
| **AL538_RS10860** | 50S ribosomal protein L16 | -0.716 | -1.463 | -1.771 | 6 |
| **AL538_RS10840** | 50S ribosomal protein L2 | -0.827 | -0.764 | -1.574 | 4 |
| **AL538_RS10850** | 50S ribosomal protein L22 | -0.954 | -2.066 | -2.065 | 6 |
| **AL538_RS10865** | 50S ribosomal protein L29 | -1.083 | -0.671 | -2.028 | 4 |
| **AL538_RS10825** | 50S ribosomal protein L3 | -1.234 | -1.966 | -2.312 | 6 |
| **AL538_RS10900** | 50S ribosomal protein L6 | 0.001 | -0.629 | -1.019 | 6 |
| **AL538_RS03580** | Endonuclease | 0.868 | 0.364 | 1.120 | 1 |
| **AL538_RS20180** | Endonuclease I | -0.445 | -0.916 | -1.033 | 3 |
| **AL538_RS04175** | Endonuclease III | -0.766 | -1.634 | -1.222 | 3 |
| **AL538_RS11800** | Ribonuclease | -1.242 | -0.500 | -1.299 | 4 |
| **AL538_RS03935** | Ribonuclease E | -0.757 | -1.385 | -0.973 | 3 |
| **AL538_RS10780** | Ribonuclease E activity regulator RraA | -1.298 | -2.066 | -2.651 | 6 |
| **AL538_RS08050** | Ribonuclease R | -0.902 | -1.215 | -1.291 | 3 |
| **AL538_RS25010** | Ribonuclease Z | 0.814 | 1.160 | 1.314 | 1 |
| **AL538_RS04915** | RNA polymerase sigma factor | -0.994 | -1.647 | -1.399 | 3 |
| **AL538_RS05025** | RNA polymerase sigma factor FliA | -0.755 | -0.910 | -1.455 | 6 |
| **AL538_RS06825** | RNA polymerase sigma factor RpoE | -0.591 | -0.781 | -1.899 | 4 |
| **AL538_RS08980** | RNA polymerase sigma factor RpoH | -0.788 | -1.491 | -1.994 | 6 |
| **AL538_RS06705** | RNA polymerase sigma factor RpoS | -1.885 | -3.163 | -3.052 | 2 |
| **AL538_RS07370** | RNA polymerase sigma-54 factor | -0.759 | -1.047 | -1.281 | 6 |
| **AL538_RS22840** | RNA polymerase subunit sigma-70 | -0.345 | -0.173 | -1.762 | 4 |
| **AL538_RS26860** | RNA polymerase subunit sigma-70 | -1.093 | -0.744 | -0.901 | 2 |
| **AL538_RS07425** | RNA polymerase-binding ATPase | -0.474 | -0.649 | -1.135 | 6 |
| **AL538_RS06965** | Deoxyribonuclease I | 0.845 | 1.282 | 1.406 | 5 |
| **Iron uptake. storage & utilization** | | | | | |
| **AL538_RS26680** | Ferric reductase | 0.938 | 1.185 | 1.140 | 5 |
| **Iron uptake/transport** | | | | | |
| **AL538_RS12515** | Fe-S cluster assembly protein SufD | 0.737 | 1.237 | 1.065 | 5 |
| **AL538_RS21155** | Iron permease | 0.959 | 1.295 | 1.249 | 1 |
| **AL538_RS06355** | Iron ABC transporter permease | 0.363 | 1.014 | 0.651 | 1 |
| **AL538_RS25685** | Iron ABC transporter | -0.667 | -0.229 | -1.637 | 4 |
| **AL538_RS23515** |  | 1.203 | 1.009 | 0.937 | 1 |
| **AL538_RS20075** |  | 1.074 | 0.899 | 1.422 | 1 |
| **AL538_RS09135** | Iron donor protein CyaY | -0.208 | -1.088 | -1.515 | 6 |
| **Iron storage** | | | | | |
| **AL538_RS07875** | Bacterioferritin | -2.859 | -2.868 | -3.496 | 2 |
| **AL538_RS12375** | Ferredoxin. 2Fe-2S type. ISC system | -0.365 | -0.256 | -1.152 | 4 |
| **Iron cluster biogenesis** | | | | | |
| **AL538_RS12520** | Bifunctional cysteine desulfurase/selenocysteine lyase | 0.660 | 0.922 | 1.077 | 5 |
| **AL538_RS16405** | Cysteine desulfurase | -2.740 | -3.362 | -3.312 | 2 |
| **AL538_RS12365** | Co-chaperone protein HscB | -0.393 | -1.289 | -0.823 | 3 |
| **Biosynthesis of L-cysteine** | | | | | |
| **AL538_RS07655** | Sulfite reductase subunit beta | -1.257 | -1.630 | -1.620 | 2 |
| **AL538_RS20130** | Sulfite reductase subunit C | 0.657 | 0.874 | 1.315 | 1 |
| **Other pathways** | | | | | |
| **Nicotinate and nicotinamide metabolism** | | | | | |
| **AL538_RS17615** | Nicotinate phosphoribosyltransferase | 0.127 | 1.043 | 0.996 | 5 |
| **AL538_RS18270** | Glutamate-aspartate symport protein | 1.114 | 1.428 | 1.169 | 5 |
| **Thiamin metabolism** | | | | | |
| **AL538_RS25360** | Thiamine biosynthesis protein ApbE | 1.047 | 1.221 | 1.312 | 1 |
| **AL538_RS11010** | Sulfate adenylyltransferase | -0.732 | -1.077 | -1.065 | 3 |
| **AL538_RS11005** | Sulfate adenylyltransferase subunit 2 | -0.987 | -1.234 | -1.033 | 2 |
| **Glutathione metabolism** | | | | | |
| **AL538_RS04180** | Lactoylglutathione lyase | -1.666 | -3.005 | -2.321 | 3 |
| **AL538_RS25390** | Glutathione S-transferase | -1.089 | -1.186 | -1.405 | 6 |
| **AL538_RS22050** |  | -1.390 | 0.111 | -0.768 | 4 |
| **AL538_RS06975** | Glutathione synthase | -1.310 | -1.297 | -1.993 | 6 |
| **Quorum sensing** | | | | | |
| **AL538_RS00010** | LuxR family transcriptional regulator | -1.042 | -1.200 | -1.147 | 2 |
| **AL538_RS06465** |  | -1.543 | -2.549 | -2.843 | 3 |
| **AL538_RS00610** | Two-component sensor histidine kinase | 0.675 | 0.721 | 1.106 | 1 |
| **AL538_RS20460** |  | 0.881 | 1.319 | 1.131 | 5 |
| **AL538_RS17990** | Two-component system sensor histidine kinase CreC | 0.864 | 0.419 | 1.090 | 1 |
| **AL538_RS24500** | Two-component system sensor histidine kinase UhpB | 0.913 | 0.719 | 1.342 | 1 |
| **AL538_RS22485** | Sensor histidine kinase | 0.822 | 1.252 | 0.725 | 5 |
| **AL538_RS19550** |  | 0.720 | 1.090 | 1.059 | 5 |
| **AL538_RS19310** |  | 1.253 | 1.080 | 1.148 | 1 |
| **AL538_RS18275** |  | 0.490 | 1.532 | 1.194 | 5 |
| **AL538_RS02980** | Hybrid sensor histidine kinase/response regulator | -0.563 | -0.792 | -1.030 | 6 |
| **AL538_RS00795** |  | 0.812 | 0.670 | 1.073 | 1 |
| **AL538_RS06770** |  | -0.218 | -0.424 | -2.037 | 4 |
| **Miscellaneous** | | | | | |
| **Stress** **response** | | | | | |
| **AL538_RS26850** | Universal stress global response regulator UspA | -1.278 | -0.854 | -1.887 | 4 |
| **AL538_RS10050** | Universal stress global response regulator UspA | -0.992 | -0.978 | -1.660 | 6 |
| **Others** | | | | | |
| **AL538_RS05435** | Outer membrane protein assembly factor BamA | -0.779 | -1.599 | -1.684 | 3 |
| **AL538_RS12420** | Outer membrane protein assembly factor BamB | -0.812 | -1.599 | -1.444 | 3 |
| **AL538_RS05240** | Outer membrane protein assembly factor BamC | -1.080 | -1.393 | -1.286 | 2 |
| **AL538_RS05720** | Outer membrane protein OmpK | -1.980 | -2.639 | -2.290 | 2 |
| **AL538_RS14325** | O-succinylbenzoate synthase | -1.153 | -1.844 | -1.224 | 2 |
| **AL538_RS22395** | 3.4-dihydroxy-2-butanone 4-phosphate synthase | 0.737 | 1.074 | 0.926 | 5 |
| **AL538_RS15695** | Biotin synthase BioB | -1.330 | -0.713 | -1.181 | 2 |
| **AL538_RS07310** | UDP-N-acetylglucosamine 1-carboxyvinyltransferase | -0.667 | -0.920 | -1.088 | 6 |
| **AL538_RS17760** | Alpha-mannosidase | 0.477 | 1.090 | 1.019 | 5 |
| **AL538_RS17770** |  | 0.790 | 1.085 | 1.166 | 5 |
| **AL538_RS17780** |  | 0.932 | 1.368 | 1.351 | 5 |
| **AL538_RS17765** |  | 1.126 | 0.913 | 1.411 | 1 |
| **AL538_RS17775** |  | 1.381 | 1.424 | 1.632 | 1 |
| **AL538_RS21700** | Beta-mannosidase | 1.160 | 1.126 | 1.271 | 1 |
| **gene.csv.7** | CsrB | -1.626 | -0.840 | -1.902 | 4 |
| **gene.csv.11** |  | -1.027 | -0.188 | -1.776 | 4 |
| **AL538_RS08095** | RNA-binding protein Hfq | -1.770 | -2.431 | -2.078 | 2 |
| **gene.csv.32** | Cold shock protein (CspA) | -0.318 | -0.419 | -1.767 | 4 |
| **gene.csv.35** |  | 1.675 | 1.647 | 1.371 | 5 |
| **Virulence factors involved in V. harveyi pathogenicity** | | | | | |
| **AL538_RS11345** | Hemolysin | -1.757 | -2.962 | -2.300 | 3 |
| **AL538_RS26170** | Heme A synthase | -0.724 | -1.094 | -1.258 | 3 |
| **AL538_RS26535** | Heme degradation protein HemS | 0.323 | 1.350 | 0.204 | 5 |
| **AL538_RS04975** | Heme exporter protein CcmB | 0.544 | 1.360 | 0.304 | 5 |
| **AL538_RS15910** | Pentaheme c-type cytochrome TorC. partial | -0.908 | -1.358 | -1.371 | 3 |
| **AL538_RS26165** | Protoheme IX farnesyltransferase | -1.184 | -1.763 | -2.012 | 3 |
| **AL538_RS23720** | Protoheme IX farnesyltransferase | 0.985 | 1.324 | 1.312 | 5 |
| **AL538_RS26540** | Putative heme utilization radical SAM enzyme HutW | 1.028 | 1.301 | 0.458 | 5 |
| **AL538_RS01310** | Sulfoxide reductase heme-binding subunit YedZ | 0.569 | 1.017 | 1.110 | 1 |
| **AL538_RS20930** | VirK protein | 0.708 | 1.912 | 1.755 | 5 |
| **AL538_RS23520** | TonB-dependent receptor | 1.039 | 1.028 | 1.106 | 1 |
| **AL538_RS16585** | TonB-dependent siderophore receptor | 1.011 | 0.645 | 0.918 | 1 |
| **AL538_RS23490** | TonB-dependent siderophore receptor | 0.791 | 1.106 | 0.958 | 5 |
| **AL538_RS01205** | TonB-dependent siderophore receptor | 0.832 | 0.827 | 1.152 | 1 |
| **AL538_RS25660** | TonB-dependent siderophore receptor | 1.338 | 1.520 | 1.380 | 5 |
| **AL538_RS24600** | Siderophore ferric iron reductase | 0.894 | 1.072 | 0.767 | 5 |
| **Chemotaxis. Motility & Biofilm** | | | | | |
| **AL538_RS19295** | Chemotaxis protein | 0.963 | 1.041 | 1.099 | 5 |
| **AL538_RS23565** |  | 1.116 | 0.788 | 1.205 | 1 |
| **AL538_RS11460** |  | 0.774 | 1.121 | 0.939 | 5 |
| **AL538_RS25425** |  | 0.892 | 1.340 | 1.235 | 5 |
| **AL538_RS21280** | Chemotaxis protein LafU | 0.997 | 1.305 | 0.946 | 5 |
| **AL538_RS10030** | Diguanylate cyclase | 0.503 | 1.289 | 0.778 | 5 |
| **AL538_RS26965** |  | 1.059 | 0.697 | 0.922 | 1 |
| **AL538_RS21410** | Diguanylate phosphodiesterase | 0.746 | 1.152 | 1.034 | 5 |
| **AL538_RS23540** |  | 0.951 | 0.980 | 1.254 | 1 |
| **AL538_RS25840** |  | 0.598 | 0.468 | 1.126 | 1 |
| **AL538_RS27365** |  | 0.825 | 1.345 | 1.024 | 5 |
| **AL538_RS13415** | Flagellar biosynthesis anti-sigma factor FlgM | -1.020 | 0.077 | -1.683 | 4 |
| **AL538_RS21335** | Flagellar biosynthesis pathway. component FlhA | 0.720 | 1.116 | 0.759 | 5 |
| **AL538_RS21340** | Flagellar biosynthesis protein FlhB | 0.891 | 1.557 | 1.256 | 5 |
| **AL538_RS21345** | Flagellar biosynthetic protein FliR | 0.808 | 1.413 | 1.439 | 5 |
| **AL538_RS21315** | Flagellar capping protein | 1.304 | 0.850 | 1.534 | 1 |
| **AL538_RS05055** | Flagellar export apparatus protein FliQ | 1.030 | 1.865 | 0.874 | 5 |
| **AL538_RS21310** | Flagellar export chaperone FliS | 1.346 | 1.556 | 1.531 | 5 |
| **AL538_RS21075** | Flagellar hook-associated protein FlgK | 1.127 | 1.588 | 0.855 | 5 |
| **AL538_RS05085** | Flagellar hook-length control protein FliK | 0.388 | 1.025 | 0.232 | 5 |
| **AL538_RS21300** | Flagellar hook-length control protein FliK | 0.463 | 1.081 | 0.755 | 5 |
| **AL538_RS24925** | Flagellar motor protein | 0.760 | 0.999 | 1.049 | 5 |
| **AL538_RS21365** | Flagellar motor switch protein FliM | 1.127 | 1.619 | 1.423 | 5 |
| **AL538_RS21385** | Flagellar M-ring protein FliF | 1.264 | 1.483 | 1.241 | 5 |
| **AL538_RS21070** | Flagellar protein | 0.834 | 1.240 | 1.240 | 5 |
| **AL538_RS21295** | Flagellar protein | 1.335 | 1.228 | 1.508 | 1 |
| **AL538_RS05165** | Flagellin | 0.446 | 1.218 | 1.041 | 5 |
| **AL538_RS04190** | Sodium-type flagellar protein MotY | 1.146 | 1.543 | 1.596 | 5 |
| **AL538_RS16835** | Pilus assembly protein | -0.034 | -1.399 | -0.470 | 3 |
| **AL538_RS01140** | Pilus assembly protein CpaB | 1.085 | 0.936 | 1.697 | 1 |
| **AL538_RS05990** | Pilus assembly protein CpaC | -0.569 | -1.303 | -1.285 | 3 |
| **AL538_RS05975** | Pilus assembly protein CpaF | -0.596 | -0.775 | -1.455 | 4 |
| **AL538_RS01120** |  | 0.939 | 1.560 | 1.340 | 5 |
| **AL538_RS25995** |  | 1.152 | 1.522 | 1.409 | 5 |
| **AL538_RS22750** | Pilus assembly protein PapD | 1.277 | 0.933 | 1.301 | 1 |
| **AL538_RS07780** | Pilus assembly protein PilN | 0.421 | 0.652 | 1.299 | 1 |
| **AL538_RS22580** | Pilus assembly protein PilZ | 0.500 | 1.024 | 0.860 | 5 |
| **AL538_RS01115** | Pilus assembly protein TadB | 0.998 | 0.673 | 1.619 | 1 |
| **AL538_RS01100** | Pilus assembly protein TadE | 0.701 | 1.752 | 1.274 | 5 |
| **Secretion** | | | | | |
| **AL538_RS01135** | General secretion pathway protein GspD | 0.912 | 0.806 | 1.300 | 1 |
| **AL538_RS10300** | General secretion pathway protein GspM | -0.116 | -0.269 | -1.203 | 2 |
| **AL538_RS01740** | Hydrophobe/amphiphile efflux-1 family RND transporter | 0.687 | 0.878 | 1.078 | 1 |
| **AL538_RS22720** |  | 0.934 | 1.207 | 0.960 | 5 |
| **Type I secretion apparatus** | | | | | |
| **AL538_RS22870** | HlyD family type I secretion periplasmic adaptor subunit | 0.673 | 1.107 | 0.793 | 5 |
| **AL538_RS00995** | Type I secretion C-terminal target domain-containing protein | -0.831 | -1.608 | -2.062 | 6 |
| **Type II secretion apparatus** | | | | | |
| **AL538_RS05980** | Type II secretion protein | -0.377 | -1.184 | -0.698 | 3 |
| **Type III secretion apparatus** | | | | | |
| **AL538_RS15295** | Translocator protein PopB | -1.081 | -1.179 | -1.296 | 2 |
| **AL538_RS15300** | Translocator protein PopD | -1.118 | -1.594 | -1.132 | 2 |
| **AL538_RS15275** | Type III secretion system regulator LcrR | 0.367 | 1.750 | 0.793 | 5 |
| **AL538_RS15125** | EscC/YscC/HrcC family type III secretion system outer membrane ring protein | 1.239 | 0.974 | 1.136 | 1 |
| **AL538_RS15130** | EscD/YscD/HrpQ family type III secretion system inner membrane ring protein | 0.795 | 1.100 | 1.732 | 1 |
| **AL538_RS15160** | EscJ/YscJ/HrcJ family type III secretion inner membrane ring protein | 1.077 | 1.212 | 1.477 | 1 |
| **AL538_RS15270** | EscV/YscV/HrcV family type III secretion system export apparatus protein | 1.152 | 1.169 | 1.261 | 1 |
| **Type IV secretion apparatus** | | | | | |
| **AL538_RS24855** | Type IV secretion protein Rhs | 0.961 | 0.747 | 1.322 | 1 |
| **Type VI secretion apparatus** | | | | | |
| **AL538_RS16980** | Type VI secretion protein | -0.970 | -3.070 | -2.631 | 3 |
| **AL538_RS16990** | Type VI secretion protein | -0.612 | -1.910 | -1.804 | 3 |
| **AL538_RS24870** | Type VI secretion protein | 1.023 | 0.684 | 1.063 | 1 |
| **AL538_RS24905** | Type VI secretion protein | 0.537 | 0.496 | 1.178 | 1 |
| **AL538_RS23415** | Type VI secretion protein | 1.769 | 1.838 | 1.741 | 5 |
| **AL538_RS16935** | Type VI secretion protein | -0.665 | -1.550 | -1.467 | 3 |
| **AL538_RS16975** | Type VI secretion protein EvpB | -1.048 | -3.869 | -2.889 | 3 |
| **AL538_RS23390** | Type VI secretion protein IcmF | 0.435 | 1.398 | 0.462 | 5 |
| **AL538_RS24920** | Type VI secretion protein IcmF | 1.105 | 0.733 | 1.124 | 1 |
| **AL538_RS16930** | Type VI secretion system-associated protein | -0.795 | -1.004 | -0.975 | 2 |
| **Drug efflux & antibiotic resistance** | | | | | |
| **AL538_RS20560** | Bcr/CflA family drug resistance efflux transporter | 0.695 | 1.286 | 1.148 | 5 |
| **AL538_RS15440** | Multidrug transporter | -0.153 | -1.599 | -0.219 | 3 |
| **AL538_RS19345** |  | 1.040 | 0.341 | 1.281 | 1 |
| **AL538_RS22455** |  | 1.175 | 1.032 | 1.447 | 1 |
| **AL538_RS20920** |  | 1.644 | 1.584 | 1.755 | 1 |
| **AL538_RS23900** | Multidrug transporter AcrB | 1.071 | 1.484 | 1.265 | 5 |
| **AL538_RS18015** | Multidrug transporter subunit MdtL | 0.553 | 1.173 | 1.141 | 5 |
| **AL538_RS22715** | MexE family multidrug efflux RND transporter periplasmic adaptor subunit | 0.948 | 0.803 | 1.183 | 1 |
| **AL538_RS22070** | Antibiotic resistance protein VanZ | -0.297 | -0.232 | -1.158 | 4 |
| **AL538_RS14540** | Penicillin-binding protein activator LpoB | -1.185 | -2.204 | -2.303 | 3 |
| **AL538_RS12170** | Penicillin-insensitive murein endopeptidase | -0.514 | -1.273 | -1.278 | 3 |
| **AL538_RS12395** | Bifunctional tRNA (adenosine(37)-C2)-methyltransferase TrmG/ribosomal RNA large subunit methyltransferase RlmN | 0.290 | 1.152 | 0.301 | 5 |
| **AL538_RS25145** | Polyketide cyclase | 0.622 | 1.104 | 1.046 | 5 |
